# Extensive Transcriptomic and Genomic Analysis Provides New Insights about Luminal Breast Cancers

**DOI:** 10.1371/journal.pone.0158259

**Published:** 2016-06-24

**Authors:** Inna Tishchenko, Heloisa Helena Milioli, Carlos Riveros, Pablo Moscato

**Affiliations:** 1 Information-based Medicine Program, Hunter Medical Research Institute, New Lambton Heights, NSW, Australia; 2 School of Electrical Engineering and Computer Science, The University of Newcastle, Callaghan, NSW, Australia; 3 School of Environmental and Life Science, The University of Newcastle, Callaghan, NSW, Australia; 4 CReDITSS Unit, Hunter Medical Research Institute, New Lambton Heights, NSW, Australia; University of Wisconsin - Madison, UNITED STATES

## Abstract

Despite constituting approximately two thirds of all breast cancers, the luminal A and B tumours are poorly classified at both clinical and molecular levels. There are contradictory reports on the nature of these subtypes: some define them as intrinsic entities, others as a continuum. With the aim of addressing these uncertainties and identifying molecular signatures of patients at risk, we conducted a comprehensive transcriptomic and genomic analysis of 2,425 luminal breast cancer samples. Our results indicate that the separation between the molecular luminal A and B subtypes—per definition—is not associated with intrinsic characteristics evident in the differentiation between other subtypes. Moreover, t-SNE and MST-*k*NN clustering approaches based on 10,000 probes, associated with luminal tumour initiation and/or development, revealed the close connections between luminal A and B tumours, with no evidence of a clear boundary between them. Thus, we considered all luminal tumours as a single heterogeneous group for analysis purposes. We first stratified luminal tumours into two *distinct* groups by their *HER2* gene cluster co-expression: *HER2-amplified luminal* and *ordinary-luminal*. The former group is associated with distinct transcriptomic and genomic profiles, and poor prognosis; it comprises approximately 8% of all luminal cases. For the remaining ordinary-luminal tumours we further identified the molecular signature correlated with disease outcomes, exhibiting an approximately continuous gene expression range from low to high risk. Thus, we employed four *virtual* quantiles to segregate the groups of patients. The clinico-pathological characteristics and ratios of genomic aberrations are concordant with the variations in gene expression profiles, hinting at a progressive staging. The comparison with the current separation into luminal A and B subtypes revealed a substantially improved survival stratification. Concluding, we suggest a review of the definition of luminal A and B subtypes. A proposition for a revisited delineation is provided in this study.

## Introduction

Approximately 70% of all breast cancers are of luminal type [1]. Luminal tumours are characterised by high levels of oestrogen (ER) and progesterone (PR) receptors, and cytokeratins (CK8 and CK18) typically observed in luminal epithelial cells [2]. Compared to luminal A, luminal B tumours are defined by higher expression of proliferation and cell cycle-related genes and lower expression of PR [3]. In 2015, the 14^th^ St. Gallen International Breast Cancer Conference revised the criterion on the identification of luminal A and luminal B (HER2-negative) subtypes [4], based on ER, PR and Ki-67 status [5, 6]; the use of histological grade for this purpose was previously declined [7]. Accordingly, all luminal B patients, determined by high levels of Ki-67, undergo anthracycline and taxane chemotherapy [8], and the HER2-positive luminal tumours, considered as luminal B [2, 9], additionally follow the anti-HER2 therapy. Overall, luminal B patients show relatively worse prognosis than luminal A, with more frequent recurrence rates and larger tumour sizes [7, 10].

While the definition of luminal A and B subtypes in clinics relies on a limited number of markers, the evaluation of transcriptomic profiles and genomic aberrations has led to a continuous refinement of the molecular classification of breast cancers [4, 11]. In the early 2000s, the pioneering analysis of microarray data employing hierarchical clustering methods defined at least four molecular breast cancer subtypes: luminal A, luminal B, HER2-enriched and basal-like [12–14]. These subtypes were associated with distinct mRNA expression profiles and clinical outcomes [14–16], and thus, considered *intrinsic*. Parker *et al*. (2009) [17] later proposed the PAM50 method: the currently most popular tool to classify tumour samples into five intrinsic subtypes, based on centroids calculated for 50 most variant genes. In this application, the distinction between luminal A and B is mainly defined by cell cycle and proliferation related genes: *ANLN*, *BIRC5*, *CEP55*, *EXO1*, *KIF2C*, *MELK*, *MKI67*, *NDC80*, *PTTG1*, *UBE2C* and *UBE2T*. An alternative to the PAM50 method, the three-gene model [18] was proposed in 2012 and employs the *AURKA* module score—also linked to cell cycle—to discriminate between the luminal subtypes.

The integrated analysis of genomic changes has revealed further complexity and diversity within cancers [19]. In particular, different breast cancer subtypes have been linked to varying copy number variations (CNVs) and aberrations (CNAs) [16]. Thus, the DNA copy number profile of luminal A, associated with low-grade tumours, has been frequently reported to display 1q gain and 16q loss. Luminal B tumours, on the other hand, show a more complex genomic pattern with additional amplifications in 8p11 (*FGFR1* locus), 8q21, 11q13, 17q12 (*HER2* locus) and 20q13, associated with therapy resistance, an increased risk of relapse and poor prognosis [3, 20–24].

In terms of the origin, there is no consensus on the placement of luminal A and B subtypes. According to the commonly employed human mammary epithelial hierarchical model, the development starting from the mammary stem cell, via luminal progenitor towards differentiated luminal cells, is considered the key path of origin for most breast cancers [25, 26]. While the basal-like carcinomas are assumed to arise from luminal progenitors, both luminal A and B are hypothesised to originate from either late luminal progenitors or differentiated luminal cells, under acquisition of stem cell-like features through acquired self-renewal mechanisms [25].

Although luminal A and B breast cancers have been perceived as distinct entities with independent oncogenic drivers, luminal cancers show high degrees of resemblance [27], are mainly separated by the proliferation state [28–30] and are considered to have similar origins [25]. This problematic also extends into practice: existing prediction methods based on molecular signature are unable to reliably classify samples into either luminal A or B subtype [31, 32], and as a consequence, to precisely define the prognosis [33]—possibly due to the presence of intermediate disease states or tissue heterogeneity [17]. In clinical applications, in addition to the previously listed reasons [34, 35], the variations in hormone receptor thresholds [7] and Ki-67 levels [36] complicate an accurate determination of luminal A or B subtypes. Several recent studies have questioned whether luminal tumours may represent a continuum, making their separation into intrinsic subtypes ambiguous [37–42]. It has also been suggested luminal A tumours may evolve into luminal B through stochastic acquisitions of mutations in genes associated with worse prognosis, including *HER2* and *TP53* [22]. Overall, luminal tumours show the highest number of mutations among patients in comparison to other subtypes [43]. This heterogeneity affects the accuracy of diagnosis and prognosis, and thus, the clinical decision-making. As a result, a profound transcriptomic and genomic characterisation of luminal breast cancers is mandatory to provide further insights into their biological definition [44, 45].

In this study, we provide a comprehensive analysis on luminal tumours, with the aim to identify factors supporting or opposing the consideration of luminal A and B subtypes as distinct entities. We also investigate the impact of the HER2 locus-amplification on luminal carcinomas. Furthermore, we identify and provide molecular signature of patients at risk, and compare it with the current separation into luminal A and B subtypes [5].

## Materials and Methods

### Data Set Descriptions

To pursue the objectives defined above, we studied the transcriptomic and genomic profiles of 2,425 luminal samples from two comprehensive data sets in breast cancer: the Molecular Taxonomy of Breast Cancer International Consortium (METABRIC) [46] and the Research Online Cancer Knowledgebase (ROCK) [47].

The complete METABRIC data set consists of over 2,000 samples (breast tumours and controls), for which the mRNA expression was measured using the Illumina HT-12 v3 platform (Illumina Human WG-v3) and the CNA using Affymetrix SNP 6.0 [46]. This data set comprises genomic and transcriptomic profiles, and information about patients’ demographic, clinico-pathological and immunohistochemical (IHC) status. It is available at the European Genome-Phenome Archive (EGA, http://www.ebi.ac.uk/ega), under the accession number EGAS00000000083. The original study collected and analysed data under the approval of the ethics Institutional Review Board [46]. The use of this data for our research was approved by the Human Ethics Research Committee (HREC) of The University of Newcastle, Australia (approval number: H-2013-0277).

Labels for five breast cancer subtypes defined using the single classifier method PAM50 [17], were also provided with the METABRIC data set. Nevertheless, for this study we employed the previously reported improved subtype labels relying on the PAM50 gene set, but appointed by means of an ensemble learning approach instead of a single classifier [32]. The resulting luminal A subtype is defined to be IHC 98% ER^+^ 4% HER2^+^, luminal B: 99% ER^+^ 12% HER2^+^, HER2-enriched: 15% ER^+^ 70% HER2^+^, and basal-like: 5% ER^+^ 3% HER2^+^. To investigate luminal tumours, we used gene expression data containing 48,803 Illumina probes for 1,360 samples labelled as luminal A or B, and 144 controls. We then randomly divided the whole METABRIC data set into two subsets of 680 samples each: training and validation (notice: these *do not* correspond to the *discovery* and *validation* sets used in the original METABRIC study [46]).

An additional independent validation data set was obtained from the ROCK interface [47]. This meta-data is based on ten different publicly available studies (GSE2034, GSE11121, GSE20194, GSE1456, GSE2603, GSE6532, GSE20437, E-TABM-185, GSE7390 and GSE5847), and can be obtained from the Gene Expression Omnibus (http://www.ncbi.nlm.nih.gov/geo), under series accession number GSE47561. It comprises 1,570 samples measured across 22,283 re-normalised probes from the Affymetrix Human Genome U133A array. In this study, we used 1,065 samples of luminal type, assigned using the same ensemble learning approach as in the METABRIC data set [32], where luminal A samples are IHC 92% ER^+^ 12% HER2^+^, luminal B are 94% ER^+^ 22% HER2^+^, HER2-enriched are 11% ER^+^ 79% HER2^+^ and basal-like are 12% ER^+^ 9% HER2^+^.

### Differential Filter

Microarray data sets usually incorporate several thousands of probes associated with varying functions and mechanisms—with more and less relevance for luminal breast cancers. Thus, we implemented a *Differential* filter and applied it to each Illumina probe, with the aim to identify those linked to luminal breast cancer initiation or/and development. This filter orders probes by their stratification power between luminal carcinomas and controls, under consideration of three cases: probe expression values in tumour samples are either (a) lower than, (b) higher than, or (c) lower *and* higher than in control samples. The last case refers to the state where a gene is dis-regulated and shows an up-regulation in some, and a down-regulation in other tumour samples. To represent the separation power of each probe, we considered the minimal *p*-value calculated using the Wilcoxon test applied to all three cases listed above. As the stratification power of these probes changes gradually, a criterion for defining a threshold was necessary. To that end we plotted the −log_10_-normalised *p*-values against the corresponding probe ranks, and defined the threshold approximately at the point where the function’s curvature is maximised.

### Separation Power between Luminal A and B

We first used the METABRIC training set to explore the relation between luminal A and B tumours with regards to the basal-like and HER2-enriched subtypes, in terms of their definition according to the PAM50 list of genes. To that end, we investigated 451 luminal A, 229 luminal B, 125 basal-like and 91 HER2-enriched tumour samples, and 144 controls. To represent the PAM50 list, we used the 48 Illumina probes previously mapped by Curtis *et al*. in the original METABRIC study [46]. To determine the separation power between two groups of samples—representing two different subtypes—we applied the non-parametric signed-rank Wilcoxon test to expression values of each probe separately. To visualise the results in form of a heat map, we applied a normalisation to each probe relative to controls, such that their resulting values range does not exceed [−1,1] and 0 corresponds to the mean expression value of controls.

There are probes over-expressed in one and under-expressed in the other molecular breast cancer subtype, with respect to controls. Under the assumption that these variations may be related to distinct underlying mechanisms associated with changes from healthy tissue to carcinomas, we examined pairs of PAM50 subtypes on the presence of such features. To that end, we used the probes, which (a) pass the Differential filter, and (b) significantly differentiate in their expression between the two subtypes. The number of probes fulfilling both conditions was defined in the same way as in the Differential filter using the ordered −log_10_-normalised Wilcoxon test *p*-values. For each probe in this subset, the mean expression value of control samples was further tested whether it is located between those corresponding to the two subtypes; probes, for which this constellation is valid, were considered as diverging in different directions from the controls.

### t-SNE and MST-*k*NN Clustering Methods

We used the Differential filter-passing probes to investigate the connections between luminal samples on a substantially larger scale than the list of genes used in the PAM50 subtyping method, with the aim to further examine the stratification of luminal carcinomas into A and B types. To that end we applied two unsupervised clustering methods: the t-Distributed Stochastic Neighbour Embedding (t-SNE) [48] and Minimum Spanning Tree-*k*-Nearest Neighbour (MST-*k*NN) [49]. The t-SNE technique was used to visualise the arrangement of luminal and control samples in the multidimensional space spanned by Illumina probes, and the MST-*k*NN graph was employed to examine the arrangement of the same samples in terms of their nearest neighbours. The MST-*k*NN tree depicts the closest relatives among samples, and thus it potentially represents the evolutionary processes of their transcriptomic divergence. In both cases we employed the square root of the Jensen-Shannon divergence [50]—a true metric—as a dissimilarity distance matrix, based on mRNA expression levels of the selected probes across all luminal tumours from the training set and the controls.

First, we computed the t-SNE mapping using the package *tsne* [51] in *R*. It maps the distance relationships between points in a multidimensional space to a two-dimensional euclidean space, by employing random walks on neighbourhood graphs. We projected the luminal and control samples to a two-dimensional space in order to obtain hints on their placement in relation to each other. To quantify the results, we drew the confidence ellipses for each sample class using the package *car* [52].

In the next stage we applied the same distance matrix in the MST-*k*NN clustering method to find natural clusters within the same population. The MST-*k*NN approach constructs a Minimum Spanning Tree connecting all samples, and then removes edges not present in the *k*-Nearest Neighbourhood graph, effectively disconnecting unrelated components. In our case the variable user-defined parameter *k* representing the number of nearest neighbours of each sample, was set to the smallest possible integer, for which the control samples are rendered in a single cluster. This condition represents the reference point where the connections between nodes within each cluster are closer or equal to those between the controls.

Further insights on the distribution of luminal A and B samples in the generated clusters were obtained by focusing on their central and peripheral regions. To that end we employed the rescaled node betweenness centrality for undirected graphs [53] to assign each node with a value reflecting the number of crossings by shortest paths between all pairs of nodes in the graph. Accordingly, the highest centrality values ranging up to 1 are assigned to nodes lying on the most “traversed” paths, while these values are equal to 0 on the leaf vertices. The threshold to separate the peripheral and central parts of the graph was set at the point where the ordered betweenness centrality values function exhibits the most curvature. These regions were then analysed on their content of samples labelled as luminal A and B by means of a binomial test, with respect to the null hypothesis that the ratio of luminal A and B nodes is the same as in the whole cohort.

We further assessed the sensitivity of the luminal A and B samples arrangement within the MST-kNN graph to the set of genes selected, by computing the same graph based on 50% and 200% of the number of probes used in the original calculation. To that end we employed the function of ordered normalised *p*-values, previously discussed in section Differential Filter, to which we applied new threshold values corresponding to a half and double of the original number of probes.

The clustering analysis described in this section was performed using an implementation of the MST-*k*NN method and the square root of the Jensen-Shannon divergence in *R* [54] and the *igraph* package [55]; the *yEd* graph editor was used for visualisation of the results.

### HER2-Amplified Luminal Tumours

The currently used PAM50 model employs centroids to determine the molecular HER2-enriched subtype predominantly consisting of ER^-^ breast cancers [17]. Nevertheless, it is known from clinical applications that some ER^+^ luminal tumours are HER2^+^. The Cancer Genome Atlas Network has also previously reported indications of the existence of at least two types of clinically defined HER2^+^ tumours with a varying expression of luminal cluster of genes including *ESR1*[43]. Thus, we determined and investigated the *HER2*-associated cluster of genes with the aim to disclose whether the current model may be biased by the presence of other genes, where two molecular types of HER2-enriched tumours—one of them corresponding to luminal carcinomas—could be considered instead.

To determine the cluster of *HER2*-related genes, we calculated the Spearman correlation between the corresponding Illumina probe and all others in the METABRIC training set. All probes with a correlation value larger than 0.5 were considered part of the cluster. Mean expression values of these genes in the HER2-enriched subtype were further used to build a characteristic centroid of a molecularly associated HER2-amplification in breast cancers, under exclusion of all other genes. This model was further used to determine HER2-amplified luminal samples, by means of the Euclidean distance.

### Survival Filter

One of the aims of this study is to identify molecular signature of luminal breast cancer patients at risk. Thus, we designed a *Survival* filter ordering genes by their association with patients’ prognosis. We employed the commonly used Kaplan-Meier estimator for survival function calculation; since this model requires lifetime data from a set of several patients, we used ordered expression values of each probe to define two corresponding groups of people with the lowest and highest levels. Two quantiles of 30% of the population (samples corresponding to the range from 0% to 30% of ordered expression values in the first, and from 70% to 100% in the second group) were selected as a trade-off between survival estimator reliability (the more samples the more robustness) and survival curves segregation capacity (the more variation in expression between the groups the more differentiation in survival curves can be achieved). The Log-Rank test was further applied to each probe to calculate a *p*-value representing its survival curves differentiation power. The ordered −log_10_-normalised *p*-values of all probes were then used to determine a threshold on the number of features passing the filter, in the same way as described in section Differential Filter, by maximising the curvature of the corresponding function. We used the package *survival* [56] in *R* to implement the filter.

### Probe Annotation

To annotate the emerging Illumina probes, we exploited the *The Database for Annotation*, *Visualization and Integrated Discovery (DAVID)* v6.7 [57, 58] containing integrated and updated source tools including GO terms, KEGG and BioCarta visualisations. To indicate significance of the appearance of a certain set of genes in a functional annotation, we used the Bonferroni corrected *p*-values calculated under consideration of the Illumina Human HT-12 V3 platform as background.

### Defining a Molecular Signature of Patients at Risk

Among the genes passing the Survival filter, there are several showing co-expression, but there are also those acting in “isolation” with only little absolute correlation to the others. Under the assumption that genes’ co-expression is a more reliable indication of a network affected by certain cell processes, and the isolated probes may represent a bias arising from the large number of total probes (48,803), we clustered the resulting data set by samples and probes. We referred to a basic hierarchical clustering methodology employing Spearman correlation as a distance matrix. To order features by their segregation power between the patients, we used two major emerging clusters of samples and the non-parametric Wilcoxon test, applied to expression values of each probe. Consequently, the top ten genes with the lowest *p*-values were selected to represent the molecular signature of patients at risk with worse prognosis.

Following the objective to separate groups within luminal tumours according to this top ten set of correlated genes with no hints to discontinuity, we used an average rank calculation to order samples from the communally lowest to highest expression levels. To be able to statistically characterise the carcinomas corresponding to varying mRNA expression levels, we introduced “subgroups”—virtually assigned groups of samples with no clear boundaries. Accordingly, we split the luminal tumours into four subgroups of the same size using quantiles; this number was chosen as a compromise between the description complexity and statistical power.

We also conducted a luminal group segregation analysis based on all probes passing the Survival filter, with the aim to assess the best possible segregation in terms of the genes selected in this study. To that end, we calculated the average rank of each sample based on these probes and ordered them to define four groups using quantiles, in an analogous way as described above.

### Validation in METABRIC and ROCK Data Sets

The Jensen-Shannon divergence metric computation for t-SNE and MST-*k*NN clustering in the METABRIC validation data set was conducted using the same list of probes selected by the Differential filter in the training set. The parameter *k* for the MST-*k*NN method was computed in the same way as in the training set. Since the METABRIC and ROCK data sets were collected using different microarray platforms, probes were mapped from Illumina to Affymetrix using the Bioconductor annotation package *illuminaHumanv3.db* [59] and *hgu133a.db* [60] in *R*, based on gene symbols. For a multiple mapping, the probe with the largest absolute expression values range in the target platform was chosen, as recommended in the *genefu* [61] package instructions.

To determine the HER2-amplified luminal tumours in the METABRIC validation set, the same centroids were used as previously calculated and applied in the training set. In the ROCK data set, the *HER2*-associated Illumina probes were first mapped to Affymetrix platform. As the centroid model is based on absolute values, these centroids were re-calculated based on 69 HER2-enriched tumours in the ROCK data set, and applied to ROCK luminal samples in an analogous way as in the METABRIC data set.

To validate the molecular signature linked to survival outcomes in the METABRIC data set, we calculated centroids of the four subgroups defined in the training set (section Defining a Molecular Signature of Patients at Risk) and applied them to the validation set, where samples were assigned to a subgroup in accordance with the shortest Euclidean distance. The segregation of samples into four subgroups in the ROCK data set was performed using the average sample ranks calculation based on Affymetrix probes mapped from the top ten Illumina set defined in the training set. Samples were ordered and divided into four equally sized groups, in the same way as described in section Defining a Molecular Signature of Patients at Risk.

### Copy Number Aberration Analysis

Following the aim to characterise genomic profiles of luminal subgroups defined in this study, we employed the original classification of CNAs in the METABRIC data set to distinguish between two categories: gains (*gains* and *amplifications*) and losses (*homozygous* and *heterozygous deletions*). We first stratified the whole genome into chromosome cytobands using the hg18 database corresponding to Illumina HT-12 v3 probes, to define DNA regions, and then calculated the occurrence rates of gains and losses on each of them. To identify the cytobands, for which the number of gains or losses significantly differs between luminal subgroups, we applied the multi-dimensional Proportion test [62]. It examines the null hypothesis that the proportion of gains/losses in each subgroup follows the global distribution of all luminal samples combined together.

To determine common genomic alterations among luminal subgroups, we overlapped the original DNA regions of gains and losses across all samples in each group separately, using the package *IRanges* [63] in *R*; this procedure yielded segregated DNA domains with their corresponding occurrence rates. We also investigated which genes may be potentially affected by or are affecting genomic transformations in luminal tumours. To that end, we used the original CNA segmentation mean data. We selected the maximal segmentation mean values within each cytoband and correlated them to the gene expression levels of survival-associated probes in the METABRIC training and validation sets separately. Only probes with correlation values larger than 0.5 and actually located on the corresponding cytobands, were selected.

## Results

### How Intrinsic Are Luminal A and B Subtypes?

#### Characterisation with Respect to PAM50 List of Genes

We studied the currently employed molecular luminal A and B subtypes with the intent to reveal how their definition and relation to each other compare to other breast cancer subtypes. To that end, we analysed the gene expression distributions of Illumina probes corresponding to the PAM50 list of genes, used for subtype determination in the METABRIC data set (S1 Table). In order to compare the separative features between luminal A and B to the differentiation between other subtypes, we additionally considered two main cases: luminal A against basal-like, and luminal A against HER2-enriched. We generated heat maps normalised with respect to control samples; this is to demonstrate where the molecular cancer subtypes are placed or have developed relative to healthy tissue.

**Fig 1:**
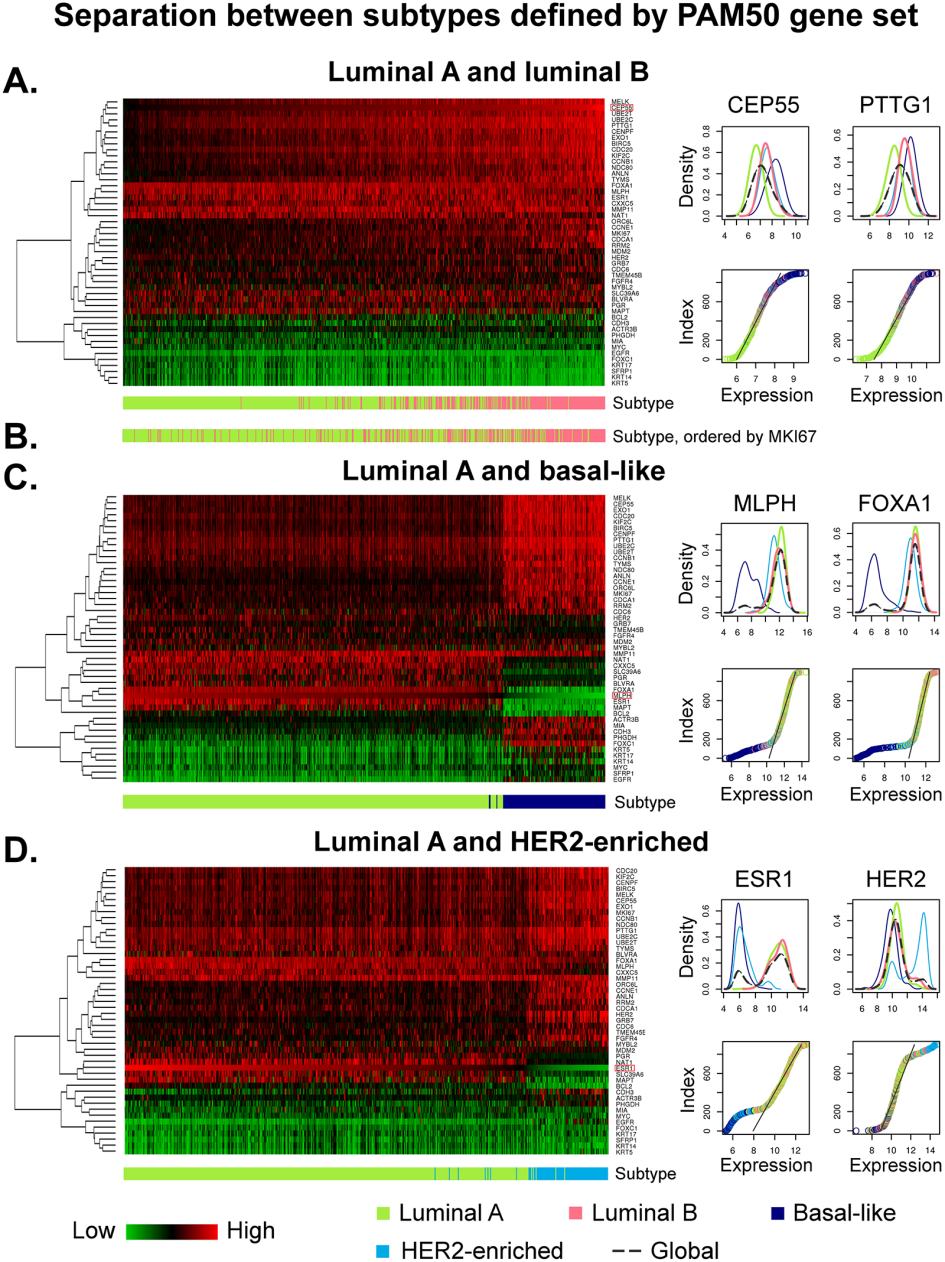
Separation features between luminal A and luminal B, basal-like, and HER2-enriched subtypes defined by PAM50 assay. The heat maps are generated from Illumina probe profiles, normalised using mean expression levels of control samples (black), where an over-expression relative to controls is shown in red, and an under-expression in green. Samples in each heat map are ordered by expression levels of the probe mostly differentiating between the corresponding pair of subtypes. (**a**) Luminal A (*n* = 451) and B (*n* = 229) samples are ordered by *CEP55*. There is no clear boundary between these subtypes evident, and the global distribution functions of the top two genes mostly differentiating between these subtypes, are unimodal. (**b**) Luminal subtypes ordered by expression levels of *MKI67*. (**c**) Luminal A (*n* = 451) and basal-like (*n* = 125) samples are ordered by absolute expression values of *MLPH*. These subtypes exhibit varying expression levels relative to controls (under- *and* over-expression), and the global density distribution functions of the top two genes are multi-modal with two peaks—one corresponding to luminal A and the other one exclusively to basal-like. (**d**) Luminal A (*n* = 451) and HER2-enriched (*n* = 91) samples are ordered by *ESR1*. These subtypes also show varying expression levels relative to controls, and the global density distribution functions of the top two probes are multi-modal.

The differentiation between luminal A and B tumours, where all samples are ordered by *CEP55*—the Illumina probe with the most separation power between these two groups—is shown in Fig 1a. This image reveals a rather gradual change of expression levels of the PAM50 genes mainly correlated to cell cycle and cell proliferation, such as *BIRC5*, *CCNB1*, *CDC20*, *CEP55*, *KIF2C*, *MELK*, *MKI67* and *UBE2C*. There is no clear boundary evident in this data set with regards to the PAM50 list, and all expression levels seem to diverge from the mean values of controls in the same direction (luminal A and B are both either under- or over-expressed for each probe). To further support this observation, we plotted density distribution functions and ordered mRNA expression values of two most representative probes of the segregation between luminal A and B subtypes (*CEP55* and *PTTG1*), also shown in Fig 1a; corresponding plots for all PAM50 genes are provided in S1 and S2 Figs. These two global density distributions are unimodal functions with only one local maximum, hinting that any separation based on thresholds, such as absolute expression levels corresponding to proliferation states, may be ambiguous. The almost linear gradient of the function representing ordered expression values, at the region where luminal A and B samples meet, also suggests an underlying unavoidable uncertainty in these groups separation based on the given set of genes.

The gene *MKI67* encoding the Ki-67 protein, recently employed for segregation between luminal A and B subtypes in clinics, is ranked as the 16th best marker separating between these two molecular subtypes, out of 48. Its relation to the luminal A and B subtypes is shown in Fig 1b, where all luminal samples are ordered by their absolute expression levels of *MKI67*. The dispersion of values across these two groups appears to be large, with no clear cut between them; nevertheless, the tendency from luminal A to luminal B concordant with the low-to-high expression change is evident.

To explore the implication of previously described results, we conducted the pairwise comparisons between luminal A, and basal-like and HER2-enriched subtypes. The relation between luminal A and basal-like tumours is illustrated in Fig 1c. All samples are ordered by *MLPH*—the top feature separating these subtypes. These two groups show expression levels diverging in different directions from the controls (relative up- and down-regulation), in several probes, including *ESR1*, *FOXC1*, *MAPT* and *MLPH*. These genes make the discrimination between luminal A and basal-like not only clearly visible, but also hint to potentially different mechanisms involved. The multimodal density distribution functions of *MLPH* and *FOXA1*—the top two separating probes—additionally affirm the presence of a boundary between luminal A and basal-like subtypes: this is the local minimum between the two maxima of the function. Ordered expression values also show the non-uniformity of their distributions among cancer samples, where basal-like are distinctly outside the nearly uniformly distributed range corresponding to luminal A and B tumours (represented by a linear gradient).

The comparison between luminal A and HER2-enriched subtypes shown in Fig 1d, also revealed rather distinct entities, where the markers *ESR1* and *HER2* (alias *ERBB2*) are main differentiators between these two molecular breast cancer subtypes. The expression values distribution functions of both markers exhibit two maxima each. These non-uniform distributions are also evident from the gradients of the functions of ordered values. The *ESR1*-associated genes are under-expressed in HER2-enriched and over-expressed in luminal A subtype, when compared to control samples; this characterisation also correlates with the IHC definition of ER being either negative or positive. The expression levels of *HER2*, on the other hand, are only elevated in HER2-enriched tumours, and ‘silenced’ in luminal A (its expression in luminal A is similar to those of controls). This observation supports the utilisation of the definition of a HER2-‘amplification’ and visualises that these two markers bear different meanings in interpretation of their ‘negative’-status with regards to control samples, or healthy tissue.

An analogous comparison between luminal B, and basal-like, and HER2-enriched subtypes is visualised in S3 Fig; it demonstrates that even though luminal B shares more similarities with basal-like and HER2-enriched subtypes than luminal A does (in terms of the PAM50 gene list)—due to high levels of expression of proliferation-related genes—the boundary between these subtypes is still clearly defined by genes being under-expressed in one and over-expressed in the other subtype, when compared to controls.

#### Variations in Expression Relative to Controls

The results described above demonstrate that the delineation of breast cancer subtypes can be correlated to diverging paths with regards to control samples, where basal-like and HER2-enriched subtypes differ from luminal A in several probes with a relative under-expression in one and an over-expression in the other group, when compared to controls. Luminal B, however, seems to be located on the same path as luminal A, where a relative under-/over-expression in the latter is also observed in the former. Assuming these variations in gene expression may be associated with different underlying mechanisms leading to alterations from healthy tissue to carcinomas, we examined whether there are probes not included in the PAM50 list of genes, with a relative under-/over-expression in luminal A and an over-/under-expression in luminal B, with respect to control samples.

Towards this goal we used the probes passing two Differential filters in a row: one segregating between luminal (A and B combined) and control samples, and the other one segregating between luminal A and B. We selected 10,000 probes, out of 48,803, passing the first Differential filter (S4 Fig); this threshold corresponds to a *p*-value of 3.93 ⋅ 10^−18^. Out of this list we further selected 1,000 probes significantly differentiating in their expression levels between the two luminal subtypes (S4 Fig); the corresponding *p*-value threshold is 1.48 ⋅ 10^−14^. From this list, only three features (*KIF13B*, *APM-1* and *DKFZP761P0423*), comprising 0.3% of all probes tested, were identified to diverge in different directions from the controls. To obtain hints on the significance of this result and to exclude bias arising from the large amount of probes used in this study, we computed the number of probes with analogous properties for the segregations between luminal A, and basal-like and HER2-enriched tumours. The differentiation between luminal A and basal-like led to an identification of 409 probes out of the top 1,000 (40.9%) with a varying expression-status (“up” or “down”) relative to controls. The comparison between luminal A and HER2-enriched resulted in 126 probes out of the top 1,000 (12.6%), up-regulated in one and down-regulated in the other subtype, when compared to control samples. All lists of probes are provided in S2 Table.

These numbers indicate that the genes associated with variations between luminal A and B samples are almost exclusively either under- or over-expressed relative to controls, in all luminal tumours, while they are diverging in basal-like and HER2-enriched subtypes when compared to luminal A. This observation additionally supports the hypothesis luminal A and B may share same cell mechanisms, resulting in similar characteristics with no clear boundary between them.

#### t-SNE Visualises Luminal and Control Samples in a Two-Dimensional Space

**Fig 2:**
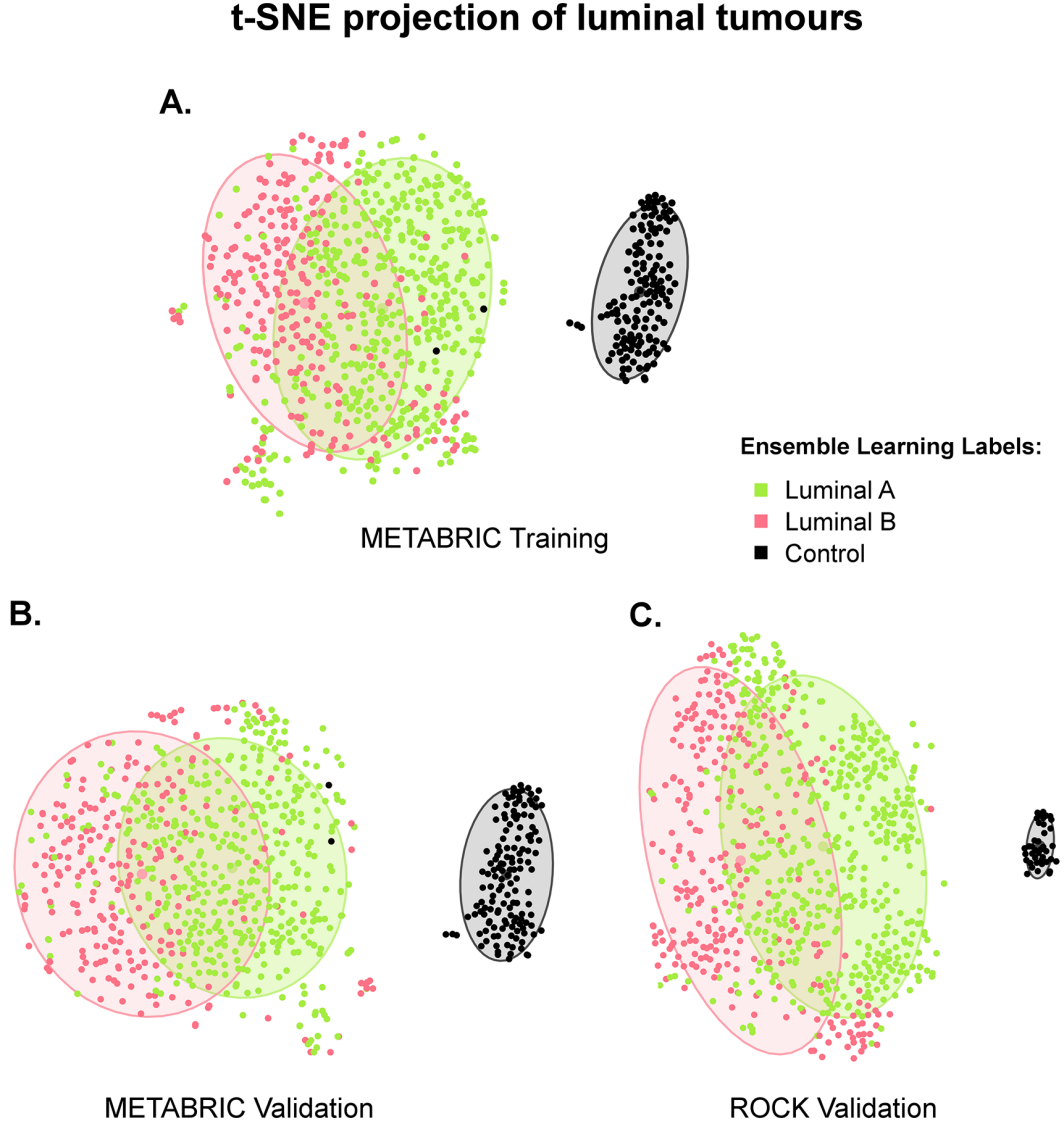
t-SNE projection of METABRIC training, validation and ROCK data sets. The t-SNE mappings to a two-dimensional space of samples from the METABRIC (**a**) training and (**b**) validation sets are based on 10,000 Illumina probes; the t-SNE computed for (**c**) the ROCK data set relies on 4,915 Affymetrix probes mapped from the Illumina platform. In all three cases there are two clusters with no overlapping between the confidence (75%) ellipses: one of them consisting of luminal A (yellow-green) and B (red), and the other one of control (black) samples. The confidence ellipses corresponding to luminal A and luminal B, however, substantially overlap each other in all three data sets. Moreover, while luminal A samples generally build compact clusters, luminal B tend to be shaped in form of a crescent around the luminal A ellipse on the opposite side from controls.

Investigations conducted in the previous paragraphs did not show any hints on the notion of luminal A and B to be independent intrinsic entities. Expanding the analysis to a larger scale, we investigated these subtypes in terms of the 10,000 previously selected probes with varying expression levels between luminal and control samples, presumably involved in the process of luminal breast cancers initiation or/and development. Thus, we mapped the 680 luminal (451 luminal A and 229 luminal B) and 144 control samples from the METABRIC training set from a 10,000- to a two-dimensional space by employing the t-SNE technique. It considers absolute distance values between multiple neighbouring nodes such that the resulting projection is a valid qualitative surrogate for the distance in the multidimensional space. The corresponding mapping is shown in Fig 2a, where the plotted ellipses represent the 75% confidence interval. These results demonstrate that the luminal (A and B combined) and control clusters are distinct, with no overlapping between their fitted confidence ellipses. The ellipses corresponding to luminal A and B tumours, however, significantly intersect each other. Moreover, while luminal A gather together in a rather compact cluster, luminal B tend to be shaped in form of a crescent around the luminal A group, where the distance to controls is maximised.

To validate these results, we applied the same methodology to the METABRIC validation (440 luminal A, 240 luminal B and 144 control samples), and ROCK (688 luminal A, 377 luminal B and 40 control samples) data sets, as shown in Fig 2b and 2c, respectively. The observations made in the training set could also be confirmed in these data sets. Accordingly, luminal A and B are similar in their molecular signature with no clear boundary between them, although there are tendencies evident that these groups have certain associations with the placement relative to each other and controls. The fact that luminal B are located around the luminal A cluster opposite the controls is indicative of their further molecular diversity from the controls than those of luminal A.

#### MST-*k*NN Clustering Reveals Connections between Luminal Tumours

**Fig 3:**
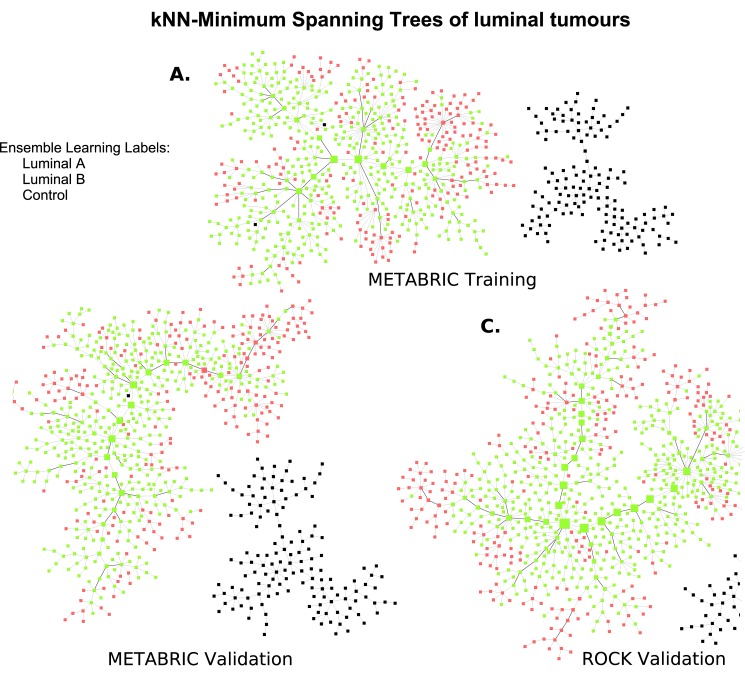
MST-4NN graphs of METABRIC training and validation sets and MST-8NN of ROCK data set. The MST-4NN graphs of samples from the METABRIC (**a**) training and (**b**) validation sets are based on 10,000 Illumina probes; the MST-8NN computed for (**c**) the ROCK data set relies on 4,915 Affymetrix probes mapped from the Illumina platform. In all three cases there are two distinct clusters, one of them consisting of luminal A (yellow-green) and B (red), and the other one of control (black) samples. By definition of the *k*-parameter, single connections between samples in the luminal cluster are as short or shorter than those between the controls. Vertex sizes are proportional to node betweenness centrality values; edges connecting the backbone of the graph are highlighted in bold lines.

To further examine the arrangement of luminal samples, we computed an MST-*k*NN clustering based on the same set of features for the METABRIC training set consisting of 680 luminal A and B samples, and 144 controls. The goal of this application is to reveal natural clusters within luminal tumours and their closest connections to each other, while considering control samples as a single group. Accordingly, the value of the parameter *k* was found to be equal to 4—the minimal integer, for which all control samples were gathered within one same cluster. The resulting graph is shown in Fig 3a. It demonstrates that all luminal tumours also got connected in a single tree, without any segregation into distinct entities. To connect the luminal with control samples, the *k*-parameter would have needed to be increased up to 20; this fact indicates the strong dissimilarity between the tumour and control samples.

While the molecular profiles of control breast tissue samples seem to be very different from those of tumours, evident from a clearly isolated entity of control samples and *k* values, the differentiation between luminal A and B subtypes is not as distinct. Within the tree, luminal A samples seem to constitute the backbone of the graph, while luminal B appear attached to and around the former. The reason for luminal A gathering together lies in their strong resemblance (represented by small divergences) between each other. Since the MST-*k*NN graph only depicts the closest neighbours, the distribution of luminal B samples on the periphery demonstrates that the individual nodes find their closest relatives among samples labelled as luminal A. This constellation indicates that the separation between these subtypes is indeterminate, emphasising the molecular similarities among luminal A and B tumours. Moreover, the conclusion can be drawn that luminal B are more heterogeneous and diverse than luminal A, evident from their location on the periphery of the tree.

To validate the observations described above, we applied the same methodology to METABRIC validation and ROCK data sets. Not all Illumina probes could be mapped to the Affymetrix platform, resulting in 4,915 target probes used for the MST-*k*NN computation in the ROCK data set. The corresponding trees are shown in Fig 3b and 3c, where the parameter *k* values were determined by the same condition as in the METABRIC training set and found to be equal to 4 and 8, respectively. In these two data sets luminal B samples also tend to appear in the periphery of the tree, around luminal A located in the central region. In order to provide a significance value of this arrangement, we applied the binomial test on the number of samples in the central part of the graph, as explained in section t-SNE and MST-*k*NN. The null hypothesis that luminal A and B samples are equally distributed in the central region as in the whole population, was rejected with *p*-values of 8.9 ⋅ 10^−7^ (4 luminal B within 61 central nodes, where the overall luminal B proportion is 33.7%), 2.8 ⋅ 10^−6^ (6 luminal B within 65 central nodes, with a reference to the overall luminal B proportion of 35.3%) and 1.9 ⋅ 10^−5^ (12 luminal B within 84 central nodes, where the overall luminal B proportion is 35.4%) for the METABRIC training and validation, and ROCK data sets, respectively. Thus, the central part of the graph is mainly comprised of luminal A tumours, while luminal B, associated with lower centrality values, are scattered near the leaves of each tree. A visualisation of the graphs segregation by luminal subtype is available in S5 and S6 Figs.

The analysis of the sensitivity of the MST-kNN graph to the number of probes selected, revealed that this luminal A and B samples arrangement is robust to perturbations. Thus, in the METABRIC training set the utilisation of 5,000 probes instead of the original 10,000 led to a *p*-value of 1.54 ⋅ 10^−5^ (8 luminal B within 72 central nodes, where the overall luminal B occurrence rate is 33.7%), indicating that the distribution of luminal A and B samples in the central region significantly diverges from those in the whole cohort. Utilising 20,000 probes instead of 10,000 also led to a significantly small *p*-value of 1.21 ⋅ 10^−5^ (5 luminal B samples within 60 central nodes, where the overall luminal B proportion is equal to 33.7%), emphasising that luminal A samples dominate the central part of the graph.

### HER2-Amplification and Luminal Tumours

Recent studies have suggested the existence of at least two subgroups within tumours with an amplification on the *HER2*-locus, expressing high or low levels of the *ESR1*, *GATA3* and *BCL2* gene cluster [43]. However, as it has been shown in the previous section of this study (Fig 1d), the current definition of the HER2-enriched subtype rather favours the identification of ER-negative tumours. Thus, we analysed all luminal tumours on the presence of a *HER2*-locus amplification, with the aim to determine whether the classification based on the PAM50 list may in some way be neglecting this group of tumours. Since the molecular HER2-enriched subtype is recognised to be more complex than a single IHC HER2-status [43], we referred to a *HER2*-associated gene cluster comprising seven probes corresponding to the genes *HER2*, *PGAP3*, *GRB7*, *STARD3*, *ORMDL3* and *MIEN1*—all located on chromosome 17 cytoband q12 (annotation and coordinates given by the hg18 database). We calculated two centroids containing absolute values of these probes for 91 HER2-enriched and 680 luminal (A or B) tumours from the METABRIC training set (Table 1), with the aim to characterise each of these entities. A further application of these centroids by means of the Euclidean distance to luminal samples in the training set led to a definition of two groups: *HER2-amplified luminal* and *ordinary-luminal*. We called the former group exhibiting an amplification on 17q12 as *HER2-amplified* to emphasise the molecular definition of the signature, and the latter was referred to as *ordinary* due to the absence of a traditionally measured HER2-amplification. These groups were further validated on the other half of the METABRIC data set using the same centroids listed in Table 1. In the ROCK data set, mapped Affymetrix probes were used to determine the HER2-amplified luminal and ordinary-luminal tumours (more details in S3 Table).

**Table 1:** Centroids for luminal and HER2-enriched subtypes.

Gene ID	Gene name	Illumina probe	Cor.	*p* -value	C_lum._	C_HER2-en._
*HER2* (*ERBB2*)	erb-b2 receptor tyrosine kinase 2	ILMN_2352131	1	0	10.65	12.86
*PGAP3* (*PERLD1*)	post-GPI attachment to proteins 3	ILMN_1805636	0.72	2.3 ⋅ 10^−144^	8.6	10.41
*GRB7*	growth factor receptor bound protein 7	ILMN_2405254	0.72	5.7 ⋅ 10^−144^	7.19	9.38
*GRB7*	growth factor receptor bound protein 7	ILMN_1740762	0.7	1.5 ⋅ 10^−132^	6.27	8.06
*STARD3*	StAR related lipid transfer domain containing 3	ILMN_1657095	0.58	1.7 ⋅ 10^−81^	6.38	7.7
*ORMDL3*	ORMDL sphingolipid biosynthesis regulator 3	ILMN_1662174	0.57	6.1 ⋅ 10^−80^	7.36	8.38
*MIEN1* (*C17orf37*)	migration and invasion enhancer 1	ILMN_1727078	0.54	3.9 ⋅ 10^−69^	6.47	7.58

The cluster of genes associated with the *HER2* expression is listed in the first column; the corresponding Illumina probe names are provided in the third and the Spearman correlation values in the fourth column; *p*-values indicating the significance of the correlation are provided in the fifth column. The absolute mean values C_lum._ and C_HER2-en._, shown in the last two columns, were calculated for luminal and HER2-enriched subtypes based on the METABRIC training set employing a log_2_-normalisation of raw data collected using the Illumina integrated system.

Patients’ clinical and demographic characteristics are listed in Table 2. The grade and NPI values are consistently significantly higher in HER2-amplified luminal breast cancers than in ordinary-luminal, and the patients tend to have been diagnosed at a slightly younger age. The tumour size, however, does not seem to correlate with the definition of these subgroups. The number of PR+ samples within the HER2-amplified cluster is significantly lower than in those without this amplification, and the occurrence rate of p53 mutation is higher. The agreement between the molecularly defined HER2-amplification and the IHC HER2-positive status is circa 85% in all three data sets. Luminal B is the most prevalent subtype within HER2-amplified luminal carcinomas with the average occurrence rate of 63%, and luminal A in ordinary-luminal with an average rate of 68%. The survival rates are significantly lower in the HER2-amplified group than in ordinary-luminal. In terms of the overall population, the HER2-amplified samples constitute a consistent amount of 7.5%-8.5% of all luminal tumours originally labelled as A or B subtypes.

**Table 2:** Clinical and demographic data for HER2-amplified luminal and ordinary-luminal patients.

	METABRIC training			METABRIC validation			ROCK		
Description	HER2-Amp.L.	Ord.-Lum.	*p* -value	HER2-Amp.L.	Ord.-Lum.	*p* -value	HER2-Amp.L.	Ord.-Lum.	*p* -value
Size [mm]	25.7	25.5	0.75	24.1	24.8	0.52	23.3 (73)	21.2 (808)	0.1
Grade	2.6	2.2	3.4 ⋅ 10^−4^	2.6	2.2	8.9 ⋅ 10^−5^	2.7 (40)	2.1 (482)	2.9 ⋅ 10^−6^
NPI	4.3	3.8	0.0055	4.2	3.8	0.006	N/A	N/A	N/A
Age at diagnosis [y.]	62.3	63.6	0.67	61.7	63.5	0.31	52.4 (72)	55.9 (772)	0.02
Lymph nodes positive	2.8	1.6	0.25	2.6	1.5	0.57	N/A	N/A	N/A
PR+/all [%]	51%	69%	0.01	45%	74%	1.7 ⋅ 10^−5^	62% (37)	80% (379)	0.016
HER2+/all [%]	84%	0%	5 ⋅ 10^−122^	86%	0%	4 ⋅ 10^−122^	86% (21)	8% (163)	5.5 ⋅ 10^−18^
Lum A/all [%]	39%	69%	4 ⋅ 10^−5^	35%	67%	1 ⋅ 10^−5^	38%	67%	5.1 ⋅ 10^−8^
P53 mutation/all [%]	25% (24)	7% (275)	0.007	26% (23)	5% (280)	8.5 ⋅ 10^−4^	N/A	N/A	N/A
10-year survival rate	0.57 (47)	0.76 (588)	0.02	0.53 (44)	0.73 (581)	5.2 ⋅ 10^−4^	0.58 (64)	0.7 (763)	0.0035
Lower limit (2.5%)	0.42	0.72		0.38	0.69		0.47	0.67	
Upper limit (97.5%)	0.77	0.81		0.74	0.78		0.72	0.74	
**Population**	**51**	**629**		**51**	**629**		**90**	**975**	

The mean values of tumour size, grade, NPI, the average numbers of positive lymph nodes, and patients’ mean age for the HER2-amplified luminal (HER2-Amp.L.) and ordinary-luminal (Ord.-Lum.) subgroups are listed in this table. The ratios of IHC PR- and HER2-positively measured cases, the prevalence of a p53 mutation, and the constitution of luminal A labels within each group, are provided as percentages. *P*-values indicating the significance of the difference between the results are also provided for each data set, where the Wilcoxon test was applied to numeric parameters and the Proportion test to Boolean parameters given as percentages. The survival rates correspond to prognostic probabilities, where 1 is the perfect prediction and 0 stands for a certain death; the lower and upper limits correspond to the 95% confidence interval of each Kaplan-Meier curve; the overall Log-rank test *p*-values of survival curves stratification are listed for each data set. In the METABRIC data set the disease-specific survival information was considered, while in the ROCK data set it corresponds to the relapse-free survival. The number of samples in each subgroup is denoted in the last row. Where the number of samples available for calculation of each characteristic was lower than 95% of the population, it is provided in parenthesis. “N/A” stands for “non-applicable” due to missing data.

**Fig 4:**
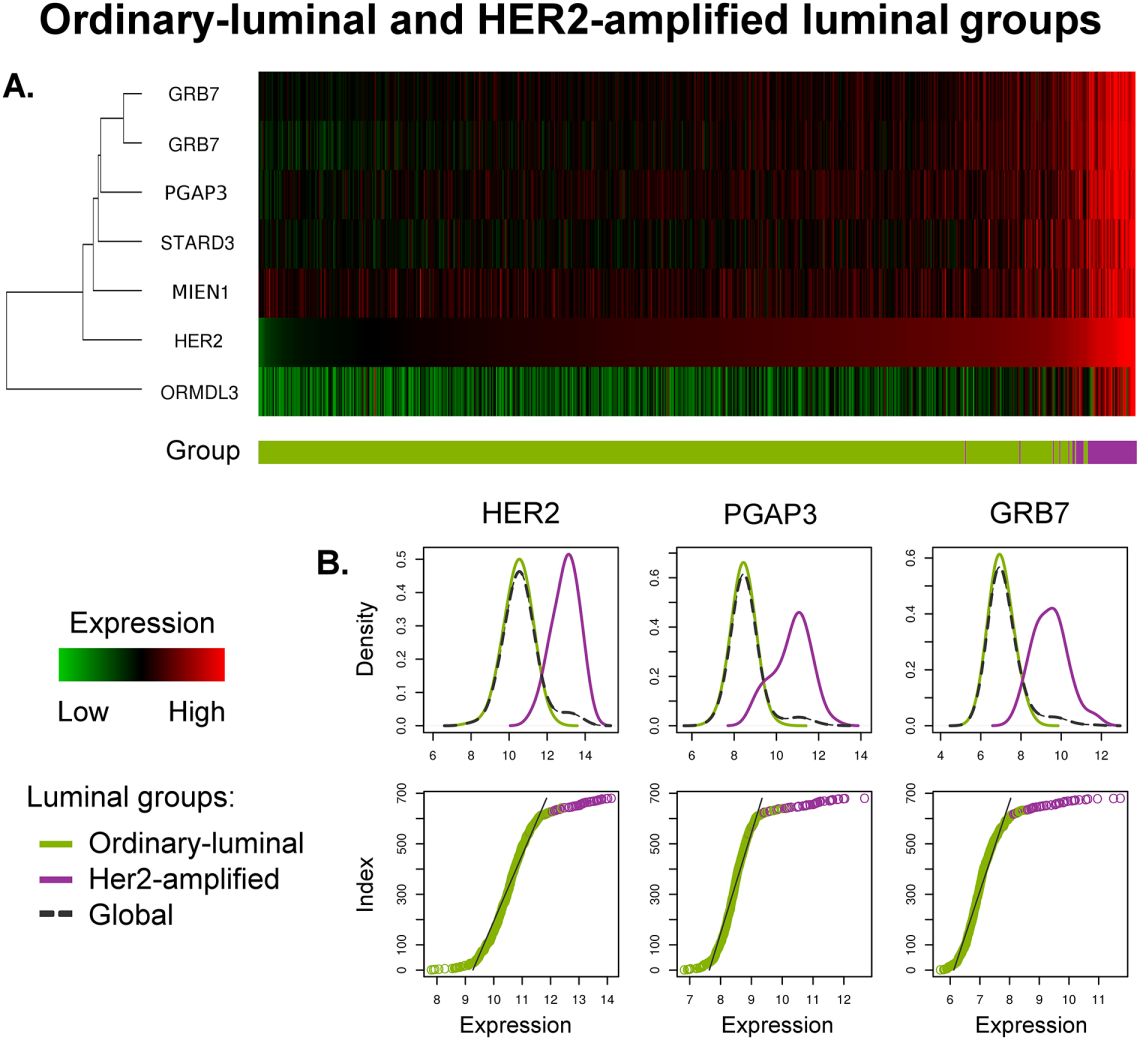
Molecular separation between ordinary-luminal and HER2-amplified luminal groups. This graph shows a comparison between ordinary-luminal (*n* = 629, shown in green), and HER2-amplified (*n* = 51, in purple) types of luminal tumours in the METABRIC training set. (**a**) The heat map is generated from Illumina probe profiles, under a normalisation with relation to the molecular signature of control samples represented by the black colour. An over-expression relative to controls corresponds to red, while an under-expression—to green. All samples are ordered by their expression levels of *HER2*. (**b**) Expression values density distributions of the top three probes correlated to and including *HER2*, are shown in the first, and ordered expression values functions—in the second row. The molecular signature of HER2-amplified is delineated by an over-expression of *HER2*-associated genes, in relation to ordinary-luminal and controls. These graphs exhibit a clearly non-uniform behaviour of probe expression levels, allowing a rather robust separation of luminal tumours.

These results demonstrate that the HER2-amplified group is an entity with consistent characteristics variant from the ordinary-luminal. To follow up this segregation in terms of the molecular signature, we plotted a heat map, shown in Fig 4a. Accordingly, the former group is associated with a well defined amplification in the subset of *HER2*-associated probes, absent in the ordinary-luminal tumours and controls. Interestingly, the expression of *ORMDL3* in ordinary-luminal is lower than the average level of control samples, while it is higher for the HER2-amplified luminal tumours. We further plotted the density distribution functions of the top three genes associated with and including *HER2*, shown in Fig 4b. These functions are multimodal. However, since the minimum density value located between the two local maxima is not well defined in each plot (the absolute difference between these values is small), this segregation is not as intrinsic as the differentiation between luminal A and basal-like subtypes (Fig 1c). On the other hand, the separation between HER2-amplified and ordinary-luminal samples is clearer than between luminal A and B (Fig 1a). The functions of ordered expression values support this conclusion (Fig 4b), where the cutting point between these two subgroups is roughly located at the break point representing the change in the nearly uniform distribution corresponding to the ordinary-luminal samples (this is the point where the approximately linear segment changes its gradient/direction). Summarising, we suggest that the separation of luminal tumours into HER2-amplified and ordinary-luminal on the molecular level, correlating to the stratification based on the IHC HER2-status [6], should be given more credence.

### Ordinary-Luminal Patients at Risk

#### Genes Associated with Prognosis

We have shown in the previous section that the HER2-amplified luminal tumours constitute approximately 8% of all luminal cases and correspond to a very poor prognosis. In this section, our aim is to identify the molecular signature of patients at risk within the remaining 92% of luminal breast cancers. To determine the genes associated with survival outcomes in ordinary-luminal patients in the METABRIC training set, we subsequently applied the Differential and Survival filters, previously described in sections Differential Filter and Survival Filter, to all Illumina probes. First, 10,000 probes were found to significantly segregate between the ordinary-luminal and controls samples; this threshold mark approximately represents the point where the curvature of ordered log_10_-normalised *p*-values is maximised, as shown in S7 Fig. Subsequently, the Survival filter was applied to each of these 10,000 probes. We further selected 600 as significantly correlated with patients’ survival probabilities based on an analogous criterion (S7 Fig). These probes can be split into four groups by their relative expression to controls (up- or down-regulation) and association with survival (positive or negative correlation): G_up-positive_, G_up-negative_, G_down-negative_ and G_down-positive_. The former two are over-expressed in tumours in relation to healthy breast tissue, where increased expression of G_up-positive_ and decreased levels of G_up-negative_ are associated with worse survival outcomes. The G_down-negative_ and G_down-positive_ clusters are delineated by an under-expression in luminal tumours compared to controls; a relative loss of expression in G_down-negative_ and an over-expression in G_down-positive_ correspond to a worse prognosis.

The functional annotation of 300 G_up-positive_ probes revealed their association with cell cycle (count: 85, *p*-value equal to 7.3 ⋅ 10^−44^), particularly with the processes of cell and nuclear division and chromosome segregation. Elevated expression levels of these probes are linked to increased cell cycle activity leading to disease progression, tumour invasiveness and worse survival outcomes. The group G_down-negative_ consisting of 178 probes, is associated with the extracellular region (count: 23, *p*-value of 0.002). Decreased levels of genes controlling the extracellular matrix can lead to a weakened adhesion capability of cancer cells, promoting cellular transformation and metastasis [64]. The mechanisms, in which the 33 probes from G_up-negative_ and 89 from G_down-positive_ are involved, remain obscure, as they are linked to a variety of functions with none of them corresponding to significant *p*-values. The complete list of all genes including their detailed functional annotation according to *DAVID* database, is provided in S4 Table.

To identify those genes from this list of 600, which are mutually co-expressed (indicating their involvement in similar mechanisms) and separate between the ordinary-luminal tumours with varying survival outcomes the most, we clustered the corresponding samples and 600 features in accordance with the methodology described in section Defining a Molecular Signature of Patients at Risk. The first ten representatives, determined by the Wilcoxon test *p*-values, from the groups G_up-positive_ (*p*-values ranging between 1.5 ⋅ 10^−72^ and 1.1 ⋅ 10^−64^) and G_down-negative_ (*p*-values ranging between 5 ⋅ 10^−49^ and 6.5 ⋅ 10^−36^) are listed in Table 3. The *p*-values of the probes in G_up-negative_ and G_down-positive_ are substantially higher (*p*-values ≥1.8 ⋅ 10^−15^ and ≥2.2 ⋅ 10^−24^ respectively), and thus, these groups are considered less significant.

**Table 3:** Top ten genes from the G_up-positive_ and G_down-negative_ groups associated with survival outcomes in ordinary-luminal patients.

Group	Gene symbol	Gene name	*p*_Survival_	*p*_Difference_
G_up-positive_	*POLQ*	polymerase (DNA directed), theta	4.6 ⋅ 10^−6^	1.5 ⋅ 10^−72^
	*CKAP2L*	cytoskeleton associated protein 2 like	6.1 ⋅ 10^−6^	8.8 ⋅ 10^−70^
	*KIFC1*	kinesin family member C1	6.2 ⋅ 10^−8^	3.4 ⋅ 10^−69^
	*FOXM1*	forkhead box M1	3.5 ⋅ 10^−8^	2.1 ⋅ 10^−68^
	*TROAP*	trophinin associated protein	9.8 ⋅ 10^−6^	8.7 ⋅ 10^−67^
	*UBE2C*	ubiquitin conjugating enzyme E2C	1.3 ⋅ 10^−6^	1.7 ⋅ 10^−66^
	*AURKB*	aurora kinase B	6.5 ⋅ 10^−5^	2.1 ⋅ 10^−66^
	*NCAPG*	non-SMC condensin I complex subunit G	1.1 ⋅ 10^−6^	3.3 ⋅ 10^−66^
	*HJURP*	Holliday junction recognition protein	1.4 ⋅ 10^−5^	5.9 ⋅ 10^−66^
	*MCM10*	minichromosome maintenance 10 replication initiation factor	2.7 ⋅ 10^−4^	1.1 ⋅ 10^−64^
G_down-negative_	*PLSCR4*	phospholipid scramblase 4	3.1 ⋅ 10^−5^	5 ⋅ 10^−49^
	*GSN*	gelsolin	3.8 ⋅ 10^−4^	2.4 ⋅ 10^−48^
	*OGN*	osteoglycin	2 ⋅ 10^−4^	2.2 ⋅ 10^−43^
	*MAMDC2*	MAM domain containing 2	4 ⋅ 10^−3^	2.6 ⋅ 10^−43^
	*DIXDC1*	DIX domain containing 1	1.2 ⋅ 10^−4^	1.4 ⋅ 10^−41^
	*CH25H*	cholesterol 25-hydroxylase	5.9 ⋅ 10^−4^	3.1 ⋅ 10^−40^
	*MIR99AHG* (*C21orf34*)	mir-99a-let-7c cluster host gene	3.9 ⋅ 10^−4^	1.2 ⋅ 10^−39^
	*CDC14B*	cell division cycle 14B	1 ⋅ 10^−4^	8.7 ⋅ 10^−38^
	*SPRY2*	sprouty RTK signaling antagonist 2	1.6 ⋅ 10^−3^	1.2 ⋅ 10^−36^
	*ANKRD35*	ankyrin repeat domain 35	2.6 ⋅ 10^−4^	6.5 ⋅ 10^−36^

The top ten genes representing each of the groups G_up-positive_ and G_down-negative_, associated with variations in survival outcomes in ordinary-luminal breast cancer patients in the METABRIC training set, are listed in the second column. G_up-positive_ genes are up-regulated in luminal tumours when compared to controls, and their over-expression is also associated with worse prognosis. G_down-negative_ are down-regulated relative to controls, and their decreased levels are correlated to lower survival rates. The Log-rank test *p*-values (*p*_Survival_) reflect the survival stratification power of each single probe based on two groups of patients of the same size (section Survival Filter). *P*-values in the last column (*p*_Difference_) were calculated using the Wilcoxon test, representing probes separation power between the clusters of luminal samples defined in section Defining a Molecular Signature of Patients at Risk.

To characterise the group G_up-positive_ in more detail, we explored the top ten set of genes on previous reports in relation to the cancer disease. The DNA polymerase theta encoded by *POLQ*, has been previously associated with radiotherapy resistance, leading to poorer prognosis in luminal breast cancers [65]. Another recent study has identified *CKAP2* as a prognostic marker for relapse-free survival in early-stage breast cancer [66]. Kinesin family member C1 encoded by *KIFC1*, plays an essential role in centrosomal bundling in cancer cells, and has been suggested as a potential therapeutic target [67, 68]. *FOXM1* has been linked to drug response, where its over-expression is correlated to apoptosis-resistant phenotype [69]. The trophinin-associated protein regulated by *TROAP*, has been correlated to aggressive clinico-pathological features including tumour high grade and mitotic rate [70], while the gene *UBE2C* has been previously recognised as a prognostic marker for high-risk breast cancer patients [71]. The expression of *AURKB* has been correlated with the level of genetic instability in lung carcinoma [72]. *HJURP* has been reported to predict the sensitivity of radiotherapy in breast cancer [73]. And an over-expression of the minichromosome maintenance 10 replication initiation factor (*MCM10*) has been suggested to be involved in the progression of cervical cancer [74].

The top ten genes from the G_down-negative_ group, including *PLSCR4*, *GSN*, *OGN*, *MAMDC2* and *DIXDC1*, *CH25H*, *MIR99AHG*, *CDC14B*, *SPRY2* and *ANKRD35*, on the other hand, are rather vaguely described in the literature. *PLSCR4* encodes phospholipid scramblase 4, a transmembrane Ca^2+^ binding protein [75]; the function of ions in relation to breast cancer has been widely discussed and it has been suggested lower levels of intracellular calcium may be preventing cell death [76]. In a recent study, the down-regulation of gelsolin—an actin-modulating protein—encoded by *GSN*, has been demonstrated to increase cell migration, leading to worse prognosis, in ER-positive breast cancer [77]. Low expression of MAM domain containing 2 encoded by *MAMDC2*, has been associated with tumour necrosis and invasion [78], while an over-expression of DIX domain containing 1 regulated by *DIXDC1*, has been shown to have a positive effect of suppressing cancer cell migration and invasion [79]. And a knock-down of the gene *SPRY2* has been previously shown to result in increased cell migration during breast morphogenesis [80].

#### Molecular Signature of Luminal Subgroups Associated with Prognosis

**Fig 5:**
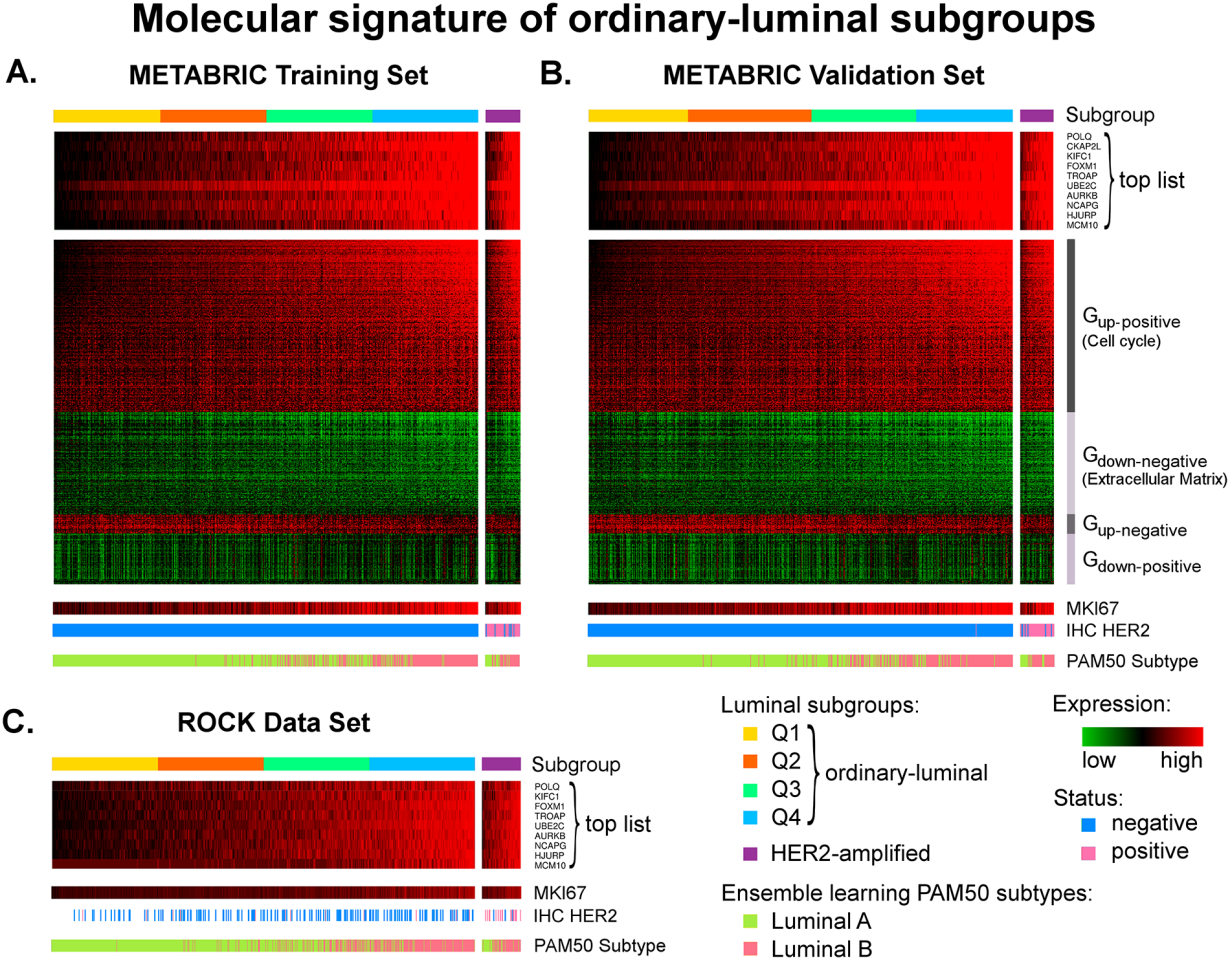
Molecular signature of ordinary-luminal subgroups. Heat maps in this figure show relative expression of the top ten list of genes used to define four quantiles within ordinary-luminal tumours: Q1 (yellow), Q2 (orange), Q3 (spring-green) and Q4 (blue), in the METABRIC training (**a**), validation (**b**) and ROCK (**c**) data sets (*n* = 629, 629 and 975, respectively). The 600 probes identified to be related to survival outcomes, split into groups G_up-positive_, G_down-negative_, G_up-negative_ and G_down-positive_, show co-expression among each other, and with the top ten list. In the METABRIC data set, the red colour corresponds to an over-expression relative to controls, green—to an under-expression, and the black colour stands for the mean expression levels of controls. In the ROCK data set, the scale black-red was used, as the controls are not available. The expression levels of same probes for the HER2-amplified luminal (*n* = 51, 51 and 90, respectively, shown in purple) groups are also shown in this figure, including the corresponding IHC HER2-status (negative in blue and positive in pink). *MKI67* expression levels and luminal A and B subtype distributions, defined using the PAM50 ensemble learning method [32], are depicted in separate bars.

Up to this point, we have analysed the ordinary-luminal breast cancers as a heterogeneous group of samples, for which certain microarray expression levels are associated with varying patients’ prognosis. With the goal to split these tumours into further subgroups based on a subset of previously defined 600 probes, we referred to the top ten genes with the lowest Wilcoxon test *p*-values from Table 3. We used these probes—all belonging to the G_up-positive_ group associated with cell proliferation state—to order luminal samples by their average rank (section Defining a Molecular Signature of Patients at Risk). To define groups within the ordinary-luminal breast cancers we referred to four quantiles, each comprising 25% of all patients, called *luminal Q1*, *Q2*, *Q3* and *Q4*; they are shown in Fig 5a. A corresponding heat map visualising expression levels of all 600 probes, previously defined to be related to varying survival rates, is also plotted in this figure. We would like to emphasise that there are no clear boundaries between the ordinary-luminal subgroups, and they are purely defined based on markers with approximately uniformly distributed expression values, for diagnostic and prognostics purposes.

Samples in the METABRIC validation set were assigned to the subgroups luminal Q1, Q2, Q3 and Q4 using the Illumina centroids calculated in the training set (Table 4). The corresponding heat maps of the top ten and all 600 probes are shown in Fig 5b. The group sizes support the existence of these molecular subgroups in terms of absolute gene expression values. These figures also show an approximately continuous transition between them. To validate ordinary-luminal subgroups in the ROCK data set, we referred to the nine Affymetrix probes mapped from the top ten set, listed in S5 Table. An analogous procedure using the average rank calculation was applied to the ROCK data set to identify four quantiles: luminal Q1, Q2, Q3 and Q4. The heat map of the top mapped genes is shown in Fig 5c; it also confirms a nearly continuous transition from patients at low to high risk. All sample IDs in the METABRIC and ROCK data sets, including their assignment to the luminal subgroups defined in this study, are provided in S6 Table.

**Table 4:** Centroids corresponding to ordinary-luminal subgroups.

Gene ID	Illumina probe	C_L.Q1_	C_L.Q2_	C_L.Q3_	C_L.Q4_
*POLQ*	ILMN_1740291	5.89	6.27	6.64	7.06
*CKAP2L*	ILMN_1751776	5.85	6.26	6.6	7.14
*KIFC1*	ILMN_2222008	6.09	6.45	6.87	7.46
*FOXM1*	ILMN_2344971	5.62	5.96	6.35	6.86
*TROAP*	ILMN_1700337	5.94	6.37	6.81	7.38
*UBE2C*	ILMN_2301083	7.54	8.50	9.15	10.06
*AURKB*	ILMN_1684217	6.07	6.50	6.91	7.58
*NCAPG*	ILMN_1751444	6.2	6.73	7.12	7.73
*HJURP*	ILMN_1703906	5.88	6.26	6.61	7.14
*MCM10*	ILMN_2413898	5.93	6.31	6.65	7.23

The absolute mean values C_L.Q1_, C_L.Q2_, C_L.Q3_ and C_L.Q4_ of the ordinary-luminal subgroups (quantiles) were calculated based on the log_2_-normalised Illumina probes listed in the second column, corresponding to the top ten genes associated with variations in survival outcomes in the METABRIC training set, shown in the first column.

#### Subgroups Characteristics

Clinico-pathological data for samples in the METABRIC training and validation sets, and in the ROCK data set, is listed in Table 5. In all data sets, tumour grade is significantly associated with gradual changes from luminal Q1 to Q4 subgroups, and consequently, with the expression levels of the top ten genes listed in Table 4. Generally, luminal Q1 carcinomas are associated with the smallest size, lowest grade and NPI, and Q4—the largest size and greatest grade and NPI. Interestingly, the significance of grade value variations among different subgroups is higher than those of NPI and the size. The ratios of IHC PR-negative and p53-mutated tumours in each group tend to increase from luminal Q1 to Q4. The survival probabilities at the 10-years mark appear in the same order as the luminal quantiles. Luminal A constitutes the luminal Q1 subgroup, while luminal B is the most frequent label in luminal Q4. These gradual changes in clinical variables going along with the mRNA expression and patients’ survival, indicate the significance of this virtual separation of ordinary-luminal breast cancers into quantiles for diagnostic and prognostic applications.

**Table 5:** Clinical and demographic data for ordinary-luminal quantiles Q1, Q2, Q3 and Q4.

	METABRIC training					METABRIC validation					ROCK				
Description	Q1	Q2	Q3	Q4	*p* -value	Q1	Q2	Q3	Q4	*p* -value	Q1	Q2	Q3	Q4	*p* -value
Size [mm]	23.1	23.2	28.7	27	6.3 ⋅ 10^−6^	21.3	24.5	25.7	27.8	6.6 ⋅ 10^−6^	20.1 (220)	20.4 (204)	21.8 (201)	22.6 (183)	0.02
Grade	1.9	2.1	2.4	2.6	1.2 ⋅ 10^−22^	1.8	2.2	2.3	2.7	2.5 ⋅ 10^−24^	1.8 (147)	2.1 (113)	2.3 (114)	2.6 (108)	5.3 ⋅ 10^−20^
NPI	3.3	3.7	4.1	4.2	4.7 ⋅ 10^−15^	3.2	3.7	3.9	4.3	5.1 ⋅ 10^−18^	N/A	N/A	N/A	N/A	N/A
Age at diagnosis [y.]	63.1	63.2	63.6	64.3	0.92	61.2	64.5	64.4	64	0.74	54.6 (171)	56.2 (195)	56.6 (204)	56.1 (202)	0.52
Lymph nodes positive	1.1	1.4	2	2.2	0.053	1.1	1.2	1.8	2.2	0.12	N/A	N/A	N/A	N/A	N/A
PR+/all [%]	76%	76%	66%	60%	0.0029	78%	84%	65%	67%	1.1 ⋅ 10^−4^	86% (85)	88% (96)	84% (81)	68% (117)	0.0011
Lum A/all [%]	100%	94%	66%	15%	2.7 ⋅ 10^−71^	100%	94%	60%	7%	3.2 ⋅ 10^−80^	100%	90%	60%	10%	2.2 ⋅ 10^−113^
P53 mutated/all [%]	4% (72)	3% (64)	9% (75)	11% (64)	0.21	2% (54)	4% (89)	3% (72)	12% (65)	0.035	N/A	N/A	N/A	N/A	N/A
10-year survival rate	0.92 (148)	0.81 (147)	0.72 (146)	0.62 (147)	2.1 ⋅ 10^−6^	0.87 (138)	0.79 (167)	0.67 (140)	0.58 (136)	5.7 ⋅ 10^−9^	0.83 (214)	0.81 (195)	0.63 (186)	0.49 (168)	3.7 ⋅ 10^−18^
Lower limit	0.87	0.74	0.64	0.54		0.81	0.71	0.59	0.5		0.76	0.75	0.56	0.41	
Upper limit	0.98	0.89	0.8	0.72		0.95	0.87	0.77	0.69		0.9	0.88	0.72	0.58	
**Population**	**158**	**157**	**157**	**157**		**147**	**183**	**155**	**144**		**244**	**244**	**244**	**243**	

The mean values of tumour size, grade, NPI, the average numbers of positive lymph nodes, and patients’ mean age for the ordinary-luminal quantiles Q1, Q2, Q3 and Q4 are listed in this table. The ratios of IHC PR- and HER2-positively measured cases, the prevalence of a p53 mutation, and the constitution of luminal A labels within each group, are provided as percentages. *P*-values indicating the significance of the difference between the results are also provided for each data set, where the multidimensional Kruskal-Wallis test (one-way ANOVA on ranks) was applied to numeric parameters and the Proportion test—to Boolean given as percentages. The survival rates correspond to prognostic probabilities, where 1 is the perfect prediction and 0 stands for a certain death; the lower and upper limits correspond to the 95% confidence interval of each Kaplan-Meier curve; the overall Log-rank test *p*-values of survival curves stratification are listed for each data set. In the METABRIC data set the disease-specific survival information was considered, while in the ROCK data set it corresponds to relapse-free survival. The number of samples in each subgroup is denoted in the last row. Where the number of samples available for calculation of each characteristic was lower than 95% of the population, it is provided in parenthesis. “N/A” stands for “non-applicable” due to missing data.

### Survival Rates Associated with *HER2*-Amplification and Proliferation Markers in Luminal Tumours

**Fig 6:**
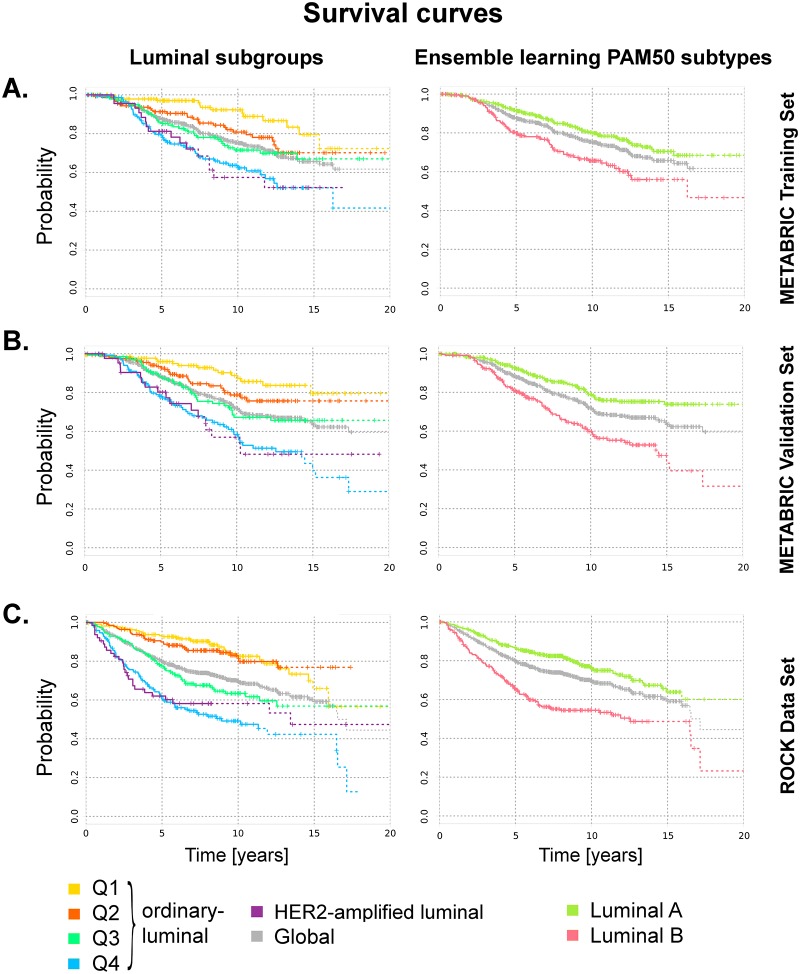
Survival curves for luminal subgroups. Survival probabilities of ordinary-luminal Q1 (yellow), Q2 (orange), Q3 (green) and Q4 (blue), and HER2-amplified luminal (purple) subgroups in the METABRIC (**a**) training, (**b**) validation and (**c**) ROCK data sets are plotted using the Kaplan-Meier estimator (*n* = 635, 625 and 827, respectively). The overall survival rates in each data set are shown in grey. Ticks represent sensors, corresponding to patients alive at a given point of time, and the drops represent deaths. The last 20 observations are denoted with a dash line. Kaplan-Meier curves of the same luminal tumours, but stratified by ensemble learning PAM50 subtypes [32], are plotted in the second column for comparison purposes. In the METABRIC data set, the disease-specific survival was considered, while in the ROCK data set the relapse-free survival was employed.

The overall survival curves of samples belonging to the ordinary-luminal Q1, Q2, Q3 and Q4, and HER2-amplified luminal groups in the METABRIC training, validation and ROCK data sets, are shown in Fig 6a, 6b and 6c, respectively. These survival probabilities show progressive changes corresponding to the probes expression values flow observed in the heat maps: luminal Q1 is associated with the best survival, while luminal Q4 shows the worst prognosis; the survival probabilities of the subgroup Q2 and Q3 are located between the former two. The prognosis of HER2-amplified luminal is very similar to those of luminal Q4 in all three data sets. The separation between these curves is highly significant: the Log-rank *p*-value is 1.2 ⋅ 10^−6^ in the METABRIC training, 3.2 ⋅ 10^−9^ in the METABRIC validation and 4.1 ⋅ 10^−18^ in the ROCK data sets. Ten year after diagnosis, the survival probabilities in the training set are 92%, 81%, 72%, 62% and 57% for luminal Q1, Q2, Q3 and Q4, and HER2-amplified luminal, respectively (Tables 2 and 5). In the METABRIC validation set these values are 87%, 79%, 67%, 58% and 52%, respectively; in the ROCK data set these are equal to 83%, 81%, 63%, 49% and 58%.

We also analysed the current separation of luminal tumours into luminal A and B subtypes for comparison purposes (Fig 6). Although the Log-rank test *p*-values are significant (5.5 ⋅ 10^−5^, 1.2 ⋅ 10^−8^ and 4.1 ⋅ 10^−13^ in the METABRIC training, validation and ROCK data sets, respectively)– not at least due to a greater number of samples in each group leading to narrower confidence intervals—this separation shows a less precise characterisation of samples than luminal quantiles defined in this study. The 10-year mark in the METABRIC training set is 80% for luminal A and 66% for luminal B; in the METABRIC validation set these values are 79% and 60%, respectively. The 10-year survival rate in the ROCK data set is 76% for luminal A and 54% for luminal B. All these values lie between those corresponding to our luminal subgroups, indicating the stratification based on the *HER2*-associated gene cluster and the top ten set of probes defined in this study provides a better segregation of luminal breast carcinomas for prognostic purposes.

It is not surprising that the survival curves of luminal Q4 show high degrees of similarity to luminal B. In the METABRIC and ROCK data sets, luminal B subtype constitutes approximately one third (33%) of all luminal tumours and is generally associated with highest expression levels of proliferation related markers. As the luminal Q4 group comprises approximately 23% of all luminal samples with the highest expression of the top ten list of genes defined in this study—also linked to proliferation state—both groups show overlaps in their samples constitution (85%, 93% and 90% of luminal Q4 samples are labelled as luminal B in the METABRIC training, validation and ROCK data sets, respectively), leading to similar characteristics.

We further assessed the survival separation power of the complete list of 600 probes passing the Survival filter by ordering the ordinary-luminal samples in accordance with their overall average ranks and splitting them into four quantiles. The resulting Kaplan-Meier curves calculated for the METABRIC training set are shown in S8 Fig. The Log-rank *p*-value of this survival curves separation is equal to 3.6 ⋅ 10^−12^; this value is lower than those corresponding to the top ten probes, and accordingly PAM50 list. Although this stratification confirms a strong association of the 600 probes selected in this study with patients’ survival, the practical implementation of this large set of biomakers in clinical setting is rather limited, not least due to the high costs associated with their joint measurement. Thus, we refer to the top ten list as the practical tool for defining the ordinary-luminal quantiles.

### CNA Aberrations Correlate to Tumourigenesis and Worse Prognosis

**Fig 7:**
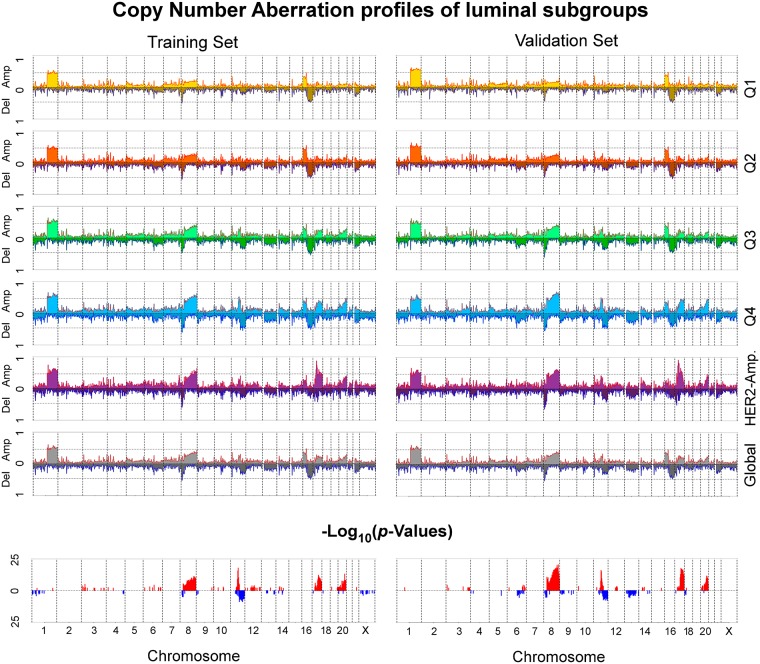
Copy Number Abberation profiles of luminal subgroups. This figure shows the CNA profiles corresponding to ordinary-luminal Q1 (yellow), Q2 (orange), Q3 (green) and Q4 (blue), and HER2-amplified luminal (purple) subgroups, in the METABRIC training and validation sets. Positive values represent gains, while negative—losses. Each bar represents a cytoband. The length of each bar corresponds to the occurrence rate within each luminal subgroup. The global CNA profile of all luminal samples combined together is shown in grey. The last row visualises the −log_10_-normalised *p*-values, calculated using the four-dimensional Proportion test, indicative for cytobands with the most variations between the ordinary-luminal (Q1, Q2, Q3 and Q4) subgroups.

The METABRIC data set—for which the CNA information is provided—was used to analyse and plot the genomic profiles of luminal groups defined above. As shown in Fig 7, there are characteristic genomic aberrations present in all luminal breast cancers, and there are those with varying occurrence rates between different subgroups. Gains on the q arm of the chromosome 1 are present in all luminal samples. Gains on the p arm of the chromosome 16 and losses on the q arm of the same chromosome occur in luminal Q1, Q2, Q3 and Q4 subgroups with similar rates, however, they are almost absent in the HER2-amplified luminal group. Furthermore, the latter group shows a clear peak on the cytoband 17q12—the *HER2*-locus—absent in the other subgroups. Genomic profiles of luminal Q1, Q2, Q3 and Q4 appear as an amplification of each other in this order; both the training and validation sets exhibit increasing rates of gains on the chromosomes 8 (cytobands 8p12, 8p11, 8q12, 8q13, 8q21, 8q22, 8q23 and 8q24), 11 (11q13), 17 (17q21, 17q22, 17q23, 17q24, 17q25) and 20 (20q13), and losses on the chromosome 11 (11q23 and 11q24). These changes are indicative for augmented levels of DNA damage in the order from luminal Q1 via Q2 and Q3 to Q4. Previous studies have also pointed to genomic aberrations on the same chromosomes linked to breast cancer tumourigenesis [81] and disease recurrence [82].

We also analysed the luminal subgroups on common CNA alterations. Remarkably, a gain on the chromosome 1 cytoband q31.3 corresponding to the location of the gene *CFH*, was found to be present in each luminal subgroup (Q1, Q2, Q3 and Q4, and HER2-amplified) with the occurrence rate of at least 50%, in both METABRIC training and validation sets. Complement factor H is generally associated with a large variety of diseases, particularly lung adenocarcinoma [83], and an over-representation of several genotypes of this gene have been previously linked to an increased risk of lung cancer and smoking [84]. Interestingly, an other recent study found smoking may lead to an increased risk of ER^+^ but not triple-negative breast cancer [85].

In this study, we also investigated which genes—from the 600 probes associated with varying survival outcomes in luminal subgroups—are linked to genomic changes. Genes, for which the corresponding probe expression levels showed a correlation to CNA segmentation mean values (as explained in section Copy Number Aberration Analysis) are listed in Table 6; all *p*-values of these correlations in both METABRIC training and validation sets range between 1.4 ⋅ 10^−44^ and 6.4 ⋅ 10^−122^. An increase in expression levels of *SDHC*, *CENPL*, *IPO9*, *ADIPOR1*, *KDM5B*, *LIN9*, *TBCE*, *RAD21*, *SQLE*, *SLC52A2*, *ZNF707*, *FAM83H*, *FBXL6*, *TRAPPC9*, *ARHGAP39*, *ADCK5*, *PRR11*, *DCAF7*, *MRPL12*, *BIRC5*, *C20orf24*, *TMEM189*, *AURKA* and *MRGBP* is associated with increased gains on the corresponding cytobands located on the chromosomes 1, 8, 17 and 20. A down-regulation of *MSRA*, on the other hand, is correlated to an increment of the amount of losses on 8p23. Several of these genes have been previously linked to tumourigenesis in breast cancer, including *KDM5B*, *RAD21*, *BIRC5*, *AURKA* and *MSRA* [86–90]. *KDM5B* has been recently shown to be over-expressed in luminal breast cancers, where high activity of this gene has also been correlated to poor outcomes in ER^+^ patients [86]. Another recent study identified *RAD21* as a driver gene within the corresponding cytoband amplification regulating the proliferation and survival of breast cancer cells, and suggested it as a potential target whose inhibition can lead to apoptosis in tumour cells [87]. *BIRC5* is a member of the inhibitor of apoptosis gene family [88], whose high expression has been previously associated with luminal B subtype [2]. A high expression of Aurora Kinase A (*AURKA*), a key regulator of chromosome segregation and cytokinesis, has been extensively associated with aggressiveness of ER^+^ breast cancer tumours and patients survival [89]. And a down-regulation of Methionine Sulfoxide Reductase A (*MSRA*) has been previously linked to a more aggressive phenotype in breast cancer [90]. Interestingly, *SLC52A2* also present in the list of genes, whose expression is associated with CNA alterations, has been previously proposed as a novel candidate blood-based marker for ovarian cancer [91].

**Table 6:** CNAs associated with gene expression in luminal tumours.

Location		Gene ID	Gene name	Group	Type	T	V
1	q23.3	*SDHC*	succinate dehydrogenase complex subunit C	G_up-pos._	Gain	✓	✓
	q25.1	*CENPL*	centromere protein L	G_up-pos._	Gain	✓	
	q32.1	*IPO9*	importin 9	G_up-pos._	Gain	✓	✓
		*ADIPOR1*	adiponectin receptor 1	G_up-pos._	Gain	✓	✓
		*KDM5B* (*JARID1B*)	lysine (K)-specific demethylase 5B	G_up-pos._	Gain	✓	✓
	q42.12	*LIN9*	lin-9 DREAM MuvB core complex component	G_up-pos._	Gain	✓	
	q42.3	*TBCE*	tubulin folding cofactor E	G_up-pos._	Gain	✓	✓
8	p23.1	*MSRA*	methionine sulfoxide reductase A	G_down-neg._	Loss	✓	
	q24.11	*RAD21*	RAD21 cohesin complex component	G_up-pos._	Gain	✓	✓
	q24.13	*SQLE*	squalene epoxidase	G_up-pos._	Gain	✓	✓
	q24.3	*SLC52A2* (*GPR172A*)	solute carrier family 52 (riboflavin transporter), member 2	G_up-pos._	Gain	✓	✓
		*ZNF707*	zinc finger protein 707	G_up-pos._	Gain	✓	✓
		*FAM83H*	family with sequence similarity 83 member H	G_up-pos._	Gain	✓	✓
		*FBXL6*	F-box and leucine-rich repeat protein 6	G_up-pos._	Gain	✓	✓
		*TRAPPC9*	trafficking protein particle complex 9	G_up-pos._	Gain	✓	✓
		*ARHGAP39* (*KIAA1688*)	Rho GTPase activating protein 39	G_up-pos._	Gain	✓	✓
		*ADCK5*	aarF domain containing kinase 5	G_up-pos._	Gain	✓	
17	q22	*PRR11*	proline rich 11	G_up-pos._	Gain		✓
	q23.3	*DCAF7* (*WDR68*)	DDB1 and CUL4 associated factor 7	G_up-pos._	Gain	✓	✓
	q25.3	*MRPL12*	mitochondrial ribosomal protein L12	G_up-pos._	Gain	✓	✓
	q25.3	*BIRC5*	baculoviral IAP repeat containing 5	G_up-pos._	Gain		✓
20	q11.23	*C20orf24*	chromosome 20 open reading frame 24	G_up-pos._	Gain	✓	✓
	q13.13	*TMEM189*	transmembrane protein 189	G_up-pos._	Gain	✓	✓
	q13.2	*AURKA*	aurora kinase A	G_up-pos._	Gain	✓	✓
	q13.33	*MRGBP* (*C20orf20*)	MRG/MORF4L binding protein	G_up-pos._	Gain	✓	✓

Genes, for which their expression is associated with CNA segmentation mean values, are provided in this table. Expression levels of the probes in the group G_up-pos._ are positively correlated to a worsening survival, while in G_down-neg._—negatively. There are two types of aberrations considered: gains and losses. The check marks indicate in which METABRIC data set (training “T” or validation “V”) the correlations between the gene expression and genomic profile were found.

## Discussion

### Luminal A and luminal B—Intrinsic Subtypes?

In this study, we have conducted a comprehensive analysis on luminal breast cancers and discussed their current stratification into luminal A and B subtypes. According to our results, luminal tumours form a heterogeneous entity and the separation into two intrinsic groups appears to be ambiguous, particularly in comparison to the clear differentiation of lumminal A (or B) from basal-like and HER2-enriched subtypes. While the termination of the basal-like subtype is delineated by non-uniformly distributed gene expression levels of *ESR1*, *MLPH*, *FOXA1*, *MAPT* and *FOXC1*, and varying molecular profiles in relation to controls (up- or down-regulation), luminal A and B do not show such intrinsic characteristics and are mainly defined by proliferation states, associated with a range of genes including *CEP55*, *MELK*, *UBE2C*, *PTTG1* and *BIRC5*. The t-SNE and MST-*k*NN clustering of luminal A and B tumours further demonstrated their close relation to each other, indicating a possible involvement of common mechanisms driving the disease outcome. In addition, luminal B samples were found to be more heterogeneous than luminal A.

Summarising, the microarray data analysis results of this study suggest that the stratification of luminal breast cancers into luminal A and B intrinsic molecular subtypes is rather arbitrary, with no conclusive evidences of underlying biological principles to be independent. Instead, the interpretation of these subtypes is consistent with a continuous variation of a molecular profile towards increasing genetic damage.

### Luminal Tumours and *HER2*-Amplification

We identified that the current definition of the molecular HER2-enriched subtype tends to favour the identification of ER-negative tumours and to neglect the ER-positives. In this study, we have demonstrated that luminal breast cancers can be separated into two molecular groups by their expression of the *HER2*-associated gene cluster located on the cytoband 17q12: HER2-amplified luminal and ordinary-luminal. The former group comprises approximately 7-8% of all luminal samples, and is delineated by the HER2+ status, high tumour grades and NPI, more frequent p53 mutations, worse survival outcomes and a slightly younger patient age, compared to the ordinary-luminal breast cancers. The genomic profile of HER2-amplified luminal samples also varies from the ordinary-luminal: it shows an outlined peak on the chromosome 17q12—the *HER2*-locus location—not present in the latter, and it does not exhibit the amount of aberrations on the chromosome 16 present in the ordinary-luminal. Interestingly, although this group is composed of up to 40% of samples originally labelled as luminal A, the survival rates of HER2-amplified luminal tumours are very low (50-60% survival chance ten years after diagnosis). These observations, in combination with a rather conclusive separation of the HER2-amplified luminal group on the molecular level, support the definition of this small aggressive group within luminal breast cancers.

### Ordinary-Luminal Breast Cancers: from Black and White to Shades of Grey

Within the remaining majority of luminal tumours (ordinary-luminal), comprising approximately 65% of all breast cancers, we identified a set of 600 probes correlated to varying prognosis. Functional annotation revealed their association with cell proliferation (up-regulation when compared to healthy tissues) and extracellular matrix ensuring cell-cell adhesion (down-regulation relative to healthy tissue). Cell cycle is commonly deregulated in breast cancer [92], and recent investigations of extracellular matrix remodelling have shown its relation to disease metastasis [64]. The top ten genes *POLQ*, *CKAP2L*, *KIFC1*, *FOXM1*, *TROAP*, *UBE2C*, *AURKB*, *NCAPG*, *HJURP* and *MCM10* were used to build a survival molecular signature, from lowest to highest risk. We further showed that samples ordered by this signature also cover a wide range of expression values and exhibit an approximately continuous flow without any clear indications towards the existence of distinct entities: ordinary-luminal tumours represent shades of grey between the extremes of black and white in terms of their molecular signature associated with survival outcomes.

To statistically characterise ordinary-luminal breast cancers corresponding to varying prognosis, we introduced four quantiles Q1, Q2, Q3 and Q4. These virtual subgroups show progressive changes in terms of gene expression, tumour grade and NPI, the number of positive lymph nodes and survival. Genomic profiles also exhibit a consistent progression from luminal Q1 to Q4, with increasing gains on the chromosomes 8, 11, 17 and 20 and losses on 11. The analysis on associations between copy number aberrations and gene expression revealed that the genes *SDHC*, *CENPL*, *IPO9*, *ADIPOR1*, *KDM5B*, *LIN9*, *TBCE*, *RAD21*, *SQLE*, *SLC52A2*, *ZNF707*, *FAM83H*, *FBXL6*, *TRAPPC9*, *ARHGAP39*, *ADCK5*, *PRR11*, *DCAF7*, *MRPL12*, *BIRC5*, *C20orf24*, *TMEM189*, *AURKA*, *MRGBP* and *MSRA* are the potential key driver candidates. While *RAD21*, *BIRC5* and *AURKA* have already been recognised as important players in luminal breast cancers progression process, the remaining genes represent novel biomarkers and targets to explore.

We further compared the performance of our stratification into quantiles (luminal Q1, Q2, Q3 and Q4) to the differentiation between luminal A and B subtypes based on the PAM50 gene set [32], with respect to survival curves segregation. Our definition provided considerably improved results, with a more precise characterisation of each group; it can be employed for diagnostic and prognostic purposes, and disease management.

## Implications

Summarising, we split all luminal tumours into two distinct groups by their HER2-amplification status (ordinary-luminal and HER2-amplified luminal), and further subdivided the ordinary-luminal group into four virtual quantiles (Q1, Q2, Q3 and Q4) using approximately uniformly distributed survival-related gene expression. Evaluating the results of this study, it seems reasonable to suppose that luminal Q4 represent an advanced stage of Q1, and possibly develop from them, while HER2-amplified luminal build a distinct entity. In the remaining section we explore implications linked to this hypothesis.

The allocation of luminal A (substantially overlapping with luminal Q1 and Q2 in this study) tumours in the human mammary epithelial hierarchical model close to differentiated luminal cells was supported by the concordance between their profile and the molecular signature of mature luminal cells [26]. Luminal B (mainly overlapping with our luminal Q4 and HER2-amplified luminal subgroups) are assumed—although inconclusively—to originate from an earlier development stage than luminal A [25, 26], possibly due to slightly lower *ESR1* expression levels, as the evolution path from a mammary stem-cell to differentiated luminal cells can be associated with changes in *ESR1* expression from low to high. Remarkably, the definition of subgroups in this study may contribute to a better understanding. The HER2-amplified luminal tumours, showing significantly lower *ESR1* expression levels than ordinary-luminal, presumably originate from an earlier development stage than luminal A (or luminal Q1/Q2). In absence of the HER2-amplification, however, the ordinary-luminal cancers are associated with higher *ESR1* expression values, with a slight increase from Q1 to Q4. This means that luminal Q3/Q4 potentially arise from a later development stage than luminal Q1/Q2, or possibly evolve from them through stochastic acquisitions of mutations due to present genomic instabilities.

It remains unclear whether luminal breast cancers can be segregated into other subtypes, not associated with different proliferation states corresponding to strongest variations in gene expression values. In this case, each subtype would be eventually represented by varying proliferation stages associated with cancer origin and/or evolution. While this segregation may be of a limited value for current clinical applications, a possible detection of such subtypes would potentially lead to a better understanding of the disease and an identification of novel targets.

We believe that a conclusive definition of molecular subtypes and their relation to each other is essential when attempting to understand the breast cancer disease. Each diverging path, or intrinsic subtype, could represent a different mechanism, and stratification based on these paths could significantly simplify the search for an appropriate model. As we show in this study, on the molecular level, both luminal A and B contain HER2-amplified cases with distinct characteristics, possibly affecting the traditional analysis results of these tumours. Thus, we suggest the recognition and separation of this particular group in future applications. Furthermore, while we endorse that a segregation based on the disease stage is beneficial for clinical prognostic purposes, following our hypothesis that ordinary-luminal breast cancers build a single heterogeneous subtype, we recommend not splitting this entity for molecular analysis, as doing so may result in discarding valuable additional information.

## Supporting Information

S1 Fig Gene expression density distributions of PAM50 list. This image shows the density distributions of 48 Illumina probes corresponding to the PAM50 genes, ordered by their separation power between the luminal A and B subtypes (from left to the right, by rows, with the most influential gene at the top left). The corresponding Wilcoxon test *p*-values are listed in S1 Table. The yellow-green line stands for luminal A, coral for luminal B, light blue for HER2-enriched, navy for basal-like and the black dashed line for all four subtypes combined together.
(TIFF)

S2 Fig Ordered expression values of PAM50 list. These graphs show mRNA expression levels of 48 Illumina probes corresponding to the PAM50 genes, plotted against the rank of each probe, ordered in the same way as in S1 Fig. The yellow-green colour stands for luminal A, coral for luminal B, light blue for HER2-enriched and navy for basal-like. The black lines are calculated based on luminal A and B samples to indicate the regions with approximately uniform distributions of expression values for these tumours.
(TIFF)

S3 Fig Separation features between luminal B, and basal-like, and HER2-enriched subtypes defined by PAM50 assay. The heat maps are generated from Illumina probe profiles, normalised using mean expression levels of control samples (black), where an over-expression relative to controls is shown in red, and an under-expression in green. Samples in each heat map are ordered by expression levels of the probe mostly differentiating between the corresponding pair of subtypes. (**a**) Luminal B (*n* = 229) and basal-like (*n* = 125) samples are ordered by *ESR1*. These subtypes exhibit varying expression levels relative to controls (under- *and* over-expression). (**b**) Luminal B (*n* = 229) and HER2-enriched (*n* = 91) samples are ordered by *ESR1*. These subtypes also show varying expression levels relative to controls.
(TIFF)

S4 Fig Wilcoxon test *p*-values distributions for comparison of tumour subtypes. These graphs show the ordered −*log*_10_ of *p*-values distributions. The first row corresponds to differentiation between controls and two tumour subtypes combined together based on all 48,803 probes from the METABRIC data set. The second row represents the separation between the two actual subtypes. The first column refers to the comparison between luminal A and B subtypes; the number of probes significantly differentiating between luminal tumours and controls was found to be equal to approximately 10,000 (red mark in the top left graph), and 1,000 probes out of the previously defined 10,000 were found to distinguish between the luminal A and B subtypes the most (red mark in the bottom left graph). The results of the comparison between luminal A and basal-like subtypes are shown in the second column. The upper graph corresponds to the separation between luminal A and basal-like tumours put together against the controls. The plot at the bottom shows the Wilcoxon test *p*-values distributions of the differentiation between luminal A and basal-like tumours. The number of probes in each graph was kept equal to those defined in luminal A and B separation: 10,000 and 1,000 respectively. The third column corresponds to the comparison between luminal A and HER2-enriched, using an analogous procedure as described above.
(TIFF)

S5 Fig Decomposition of MST-*k*NN by subtype: luminal A and controls. This graph visualises the decomposition of the MST-4NN computed for the METABRIC training and validation sets and the MST-8NN of the ROCK data set, by luminal subtype. The backbone of the tree is shown in bold. All luminal A tumours are painted in yellow-green.
(EPS)

S6 Fig Decomposition of MST-*k*NN by subtype: luminal B and controls. This graph visualises the decomposition of the MST-4NN computed for the METABRIC training and validation sets and the MST-8NN of the ROCK data set, by luminal subtype. The backbone of the tree is shown in bold. All luminal B tumours are painted in coral.
(EPS)

S7 Fig Wilcoxon test *p*-values distributions for ordinary-luminal tumours. The graph on the left shows the distribution of −log_10_-normalised *p*-values, calculated using the Wilcoxon test comparing the expression of ordinary-luminal tumours to control samples. The distribution of *p*-values calculated using the Log-rank test applied to Kaplan-Meier survival curves, as explained in section Survival Filter, is plotted on the right.
(TIFF)

S8 Fig Survival curves of luminal groups stratified based on 600 survival-related probes. The disease-specific survival probabilities of ordinary-luminal Q1 (yellow), Q2 (orange), Q3 (green) and Q4 (blue), and HER2-amplified luminal (purple) subgroups, stratified based on 600 Survival filter passing probes, in the METABRIC training set (*n* = 635) are plotted using the Kaplan-Meier estimator. The overall survival rates are shown in grey. Ticks represent sensors, corresponding to patients alive at a given point of time, and the drops represent deaths. The last 20 observations are denoted with a dash line.
(TIFF)

S1 Table The list of Illumina probes corresponding to PAM50 list. For each probe, the Wilcoxon test *p*-value reflecting the separation power between the luminal A and B subtypes, is listed in the fourth column. The absolute difference in mean log_2_-normalised gene expression values between these subtypes is provided in the fifth column. The density distribution function type is listed in the last column, based on the graphs shown in S1 Fig. Uni-modal functions exhibit only one maximum, while multi-modal show at least two distinct local maxima.
(XLS)

S2 Table The lists of Illumina probes mostly separating between luminal A and luminal B, basal-like and HER2-enriched subtypes. There are 3 tables provided in this file. The first data sheet corresponds to the analysis on luminal A and B subtypes. The first list of 10,000 probes was determined by means of Wilcoxon test *p*-values of the segregation between luminal A and B tumours combined together and controls. The second set of 1,000 out of the previously selected 10,000 probes, corresponds to those with the most separation power between luminal A and B subtypes. The last list (of 3 entities) reflects the genes, for which the mean expression of controls is located between those corresponding to luminal A and luminal B. The second data sheet has the same structure, but using basal-like tumours instead of luminal B. In the third table the results from an analogous analysis are provided, where the HER2-enriched subtype was employed instead of luminal B.
(XLS)

S3 Table Centroid values of *HER2*-associated gene cluster for luminal and HER2-enriched subtypes in the ROCK data set. The three Affymetrix probes, which could be mapped from six *HER2*-associated unique genes listed in Table 1, and their absolute mean values corresponding to the HER2-enriched and luminal subtypes are provided in this table.
(XLS)

S4 Table List of probes associated with survival outcomes and their functional annotation. The list of 600 probes mostly associated with survival outcomes of ordinary-luminal patients is provided in the first data sheet. There are four types of genes: (**1**) the “up-positive” are over-expressed in ordinary-luminal patients compared to controls and their elevated levels are also correlated to worst prognosis; (**2**) “down-negative” genes show lower expression levels in luminal tumours than in controls and their decreased levels are associated with worse survival outcomes; (**3**) “up-negative” are over-expressed in ordinary-luminal samples compared to controls, however, their under-expression is associated with worse prognosis; (**4**) and “down-positive” are under-expressed in luminal tumour samples in comparison to controls, however, their elevated levels correspond to worse survival. The Log-rank survival test *p*-values indicating the significance of the stratification of patients into two groups of the same size by expression values of each probe, are listed in the fourth column. The Wilcoxon test *p*-values arising from the comparison between two major groups of ordinary-luminal tumours emerging from the hierarchical clustering, are listed in the last column. The functional annotation of each group of genes provided by *DAVID* is included in the sheets 2–5.
(XLS)

S5 Table List of Affymetrix probes defining the molecular signature of patients at risk. The list of nine Affymetrix probes mapped from the top ten genes defined in the Illumina platform, delineating the molecular signature of patients at risk, is provided in this table.
(XLS)

S6 Table Samples assignment to luminal subgroups in the METABRIC and ROCK data sets. The list of 680 luminal samples in the METABRIC training, 680 in the METABRIC validation, and 1,065 in the ROCK data sets, including their assignment to the luminal subgroups defined in this study (ordinary-luminal quantiles Q1, Q2, Q3 and Q4, and HER2-amplified luminal), are provided in this file.
(XLS)
