# Supplementary figures and images for: Extensive Transcriptomic and Genomic Analysis Provides New Insights about Luminal Breast Cancers
Source: PLoS One. 2016 Jun 24;11(6):e0158259. doi: 10.1371/journal.pone.0158259 (PMC4920434; doi:10.1371/journal.pone.0158259)

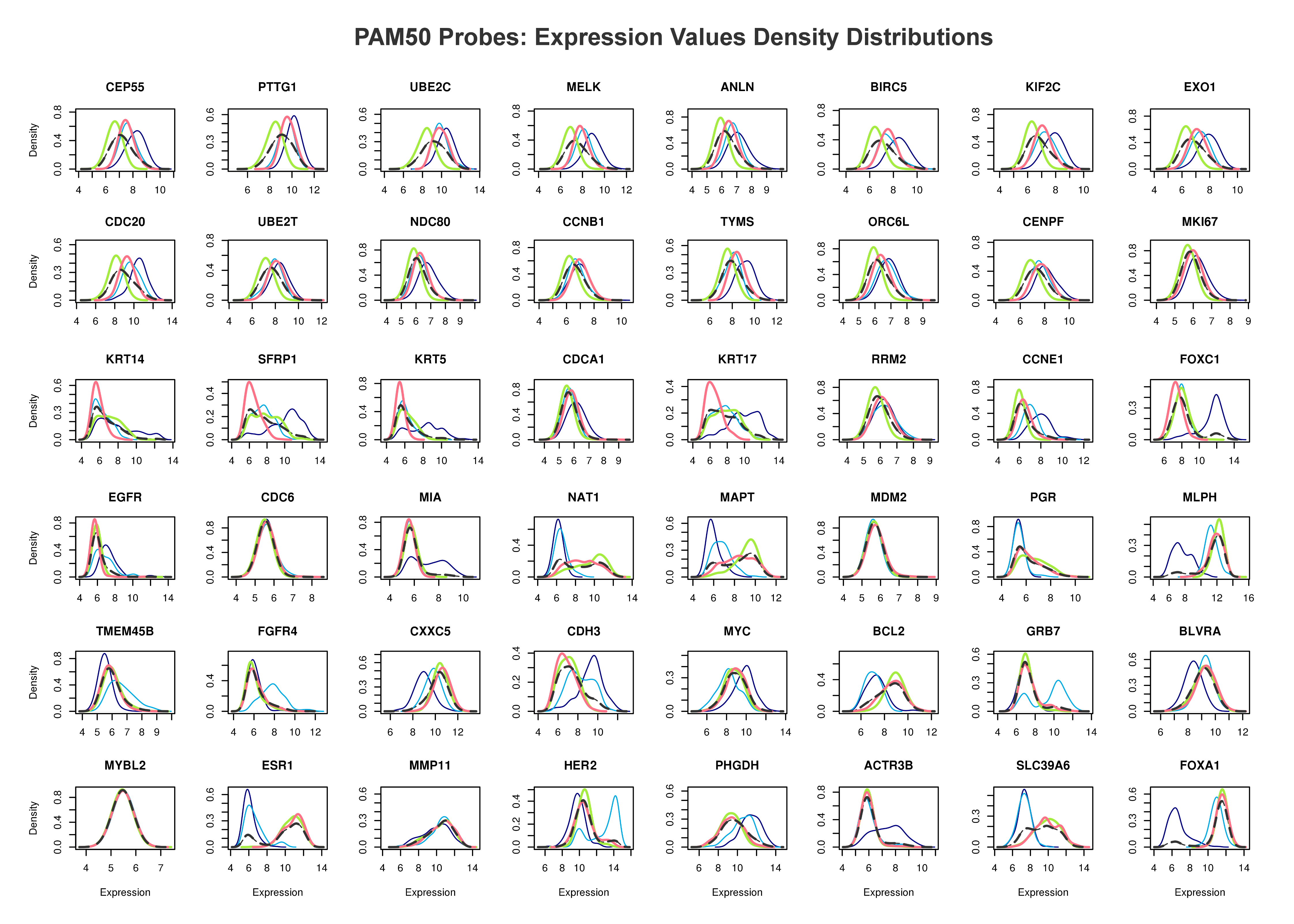

Supplement: S1 Fig — This image shows the density distributions of 48 Illumina probes corresponding to the PAM50 genes, ordered by their separation power between the luminal A and B subtypes (from left to the right, by rows, with the most influential gene at the top left). The corresponding Wilcoxon test p-values are listed in S1 Table. The yellow-green line stands for luminal A, coral for luminal B, light blue for HER2-enriched, navy for basal-like and the black dashed line for all four subtypes combined together. (TIFF) [file pone.0158259.s001.tiff]

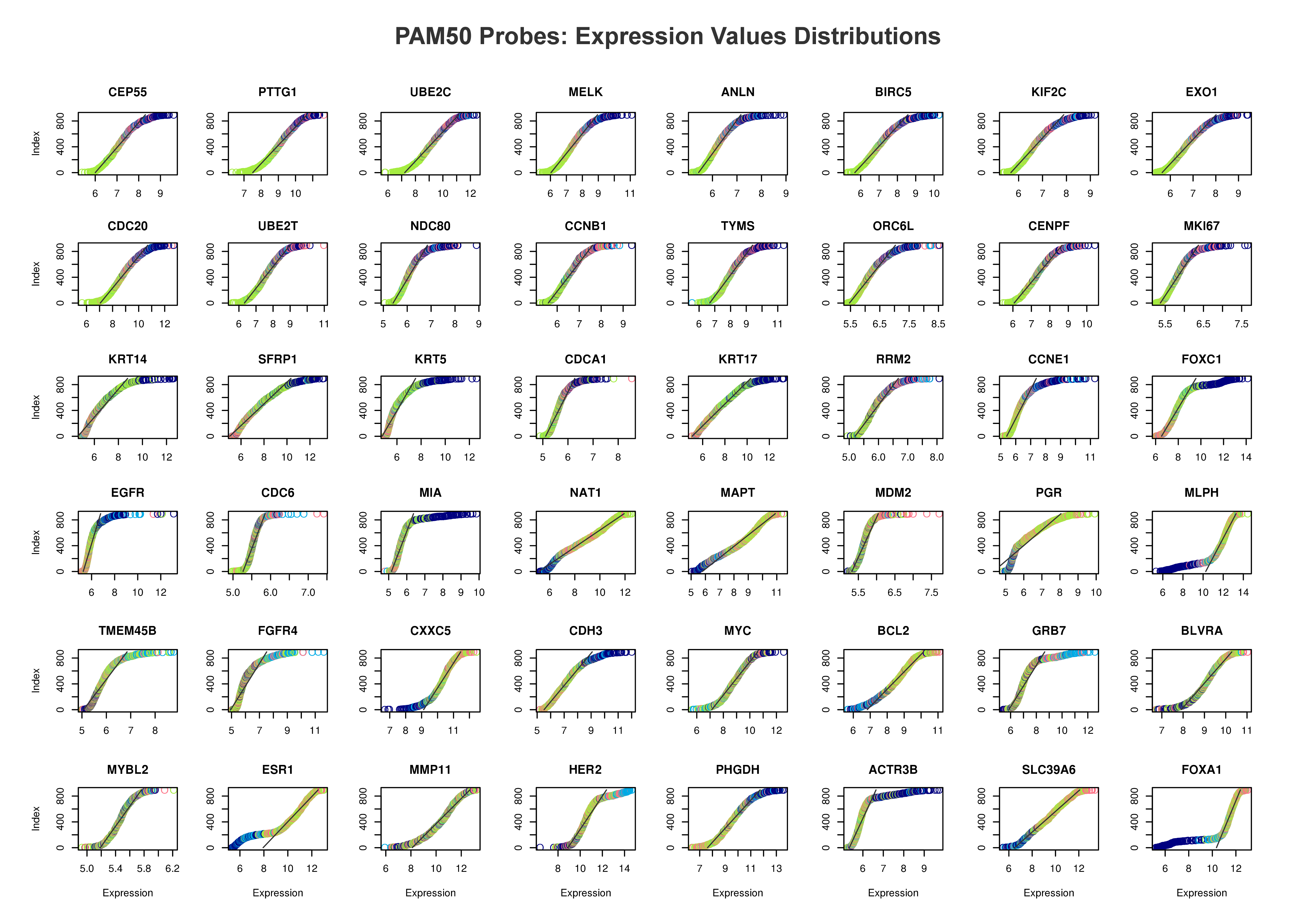

Supplement: S2 Fig — These graphs show mRNA expression levels of 48 Illumina probes corresponding to the PAM50 genes, plotted against the rank of each probe, ordered in the same way as in S1 Fig. The yellow-green colour stands for luminal A, coral for luminal B, light blue for HER2-enriched and navy for basal-like. The black lines are calculated based on luminal A and B samples to indicate the regions with approximately uniform distributions of expression values for these tumours. (TIFF) [file pone.0158259.s002.tiff]

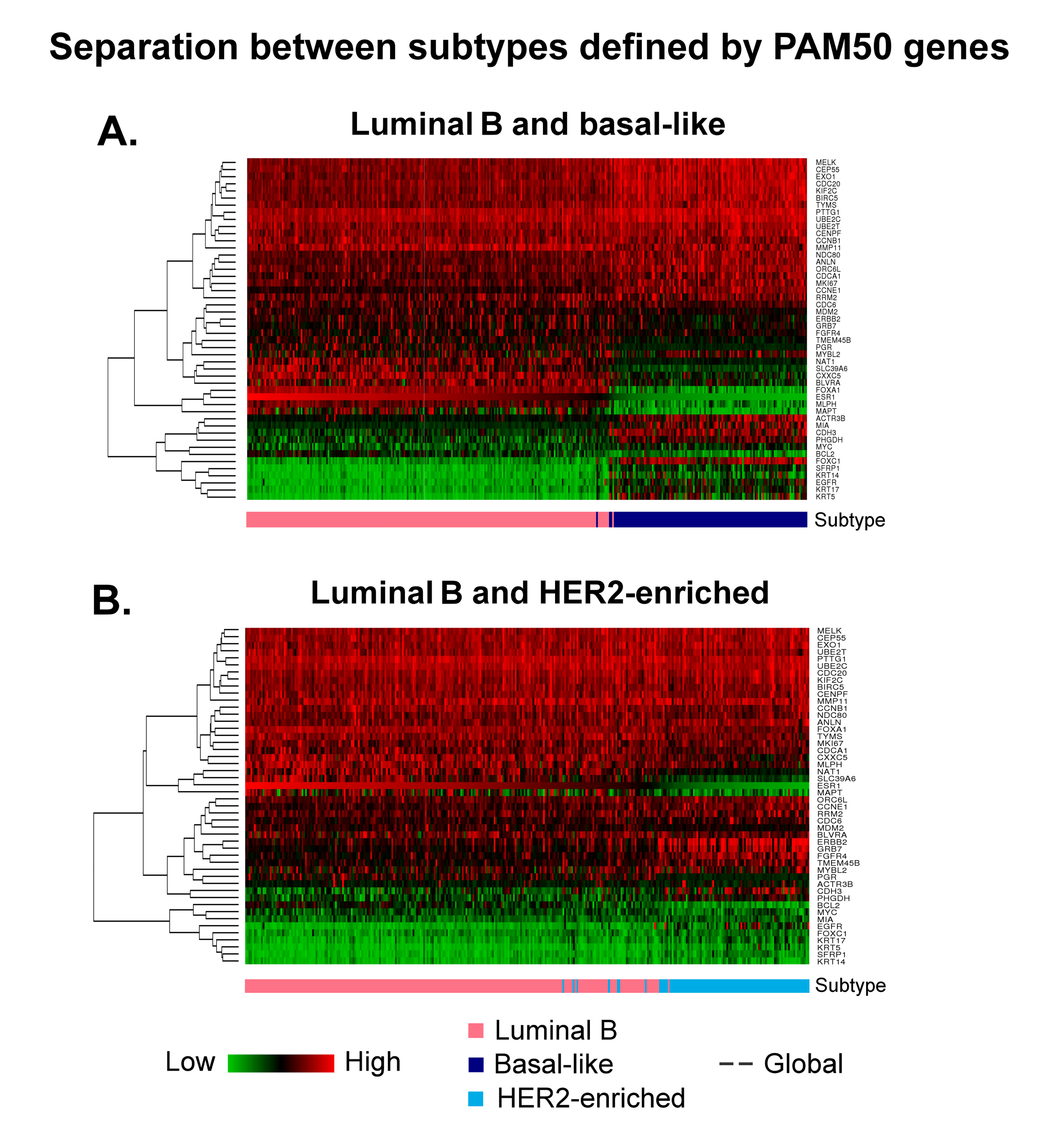

Supplement: S3 Fig — The heat maps are generated from Illumina probe profiles, normalised using mean expression levels of control samples (black), where an over-expression relative to controls is shown in red, and an under-expression in green. Samples in each heat map are ordered by expression levels of the probe mostly differentiating between the corresponding pair of subtypes. (a) Luminal B (n = 229) and basal-like (n = 125) samples are ordered by ESR1. These subtypes exhibit varying expression levels relative to controls (under- and over-expression). (b) Luminal B (n = 229) and HER2-enriched (n = 91) samples are ordered by ESR1. These subtypes also show varying expression levels relative to controls. (TIFF) [file pone.0158259.s003.tiff]

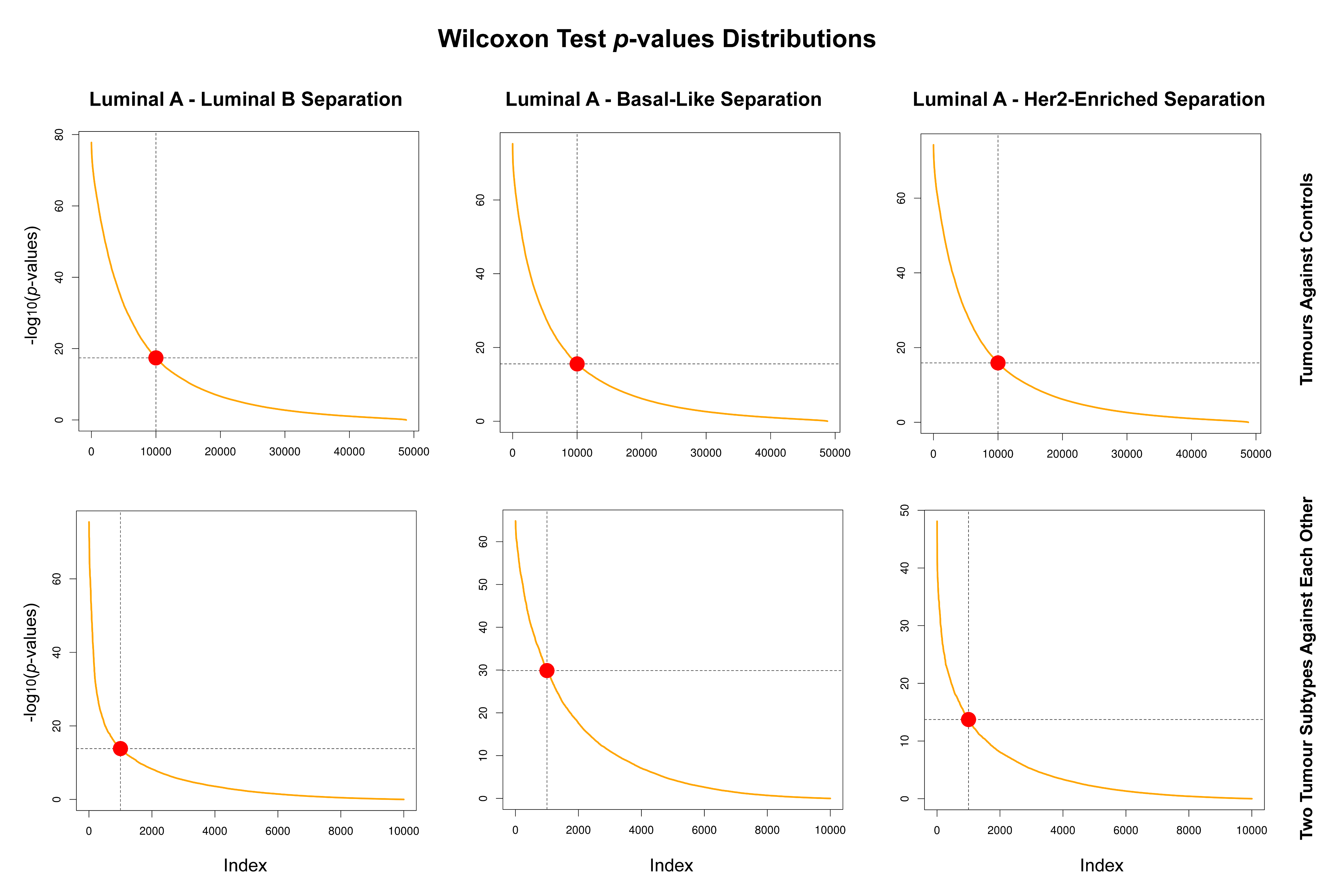

Supplement: S4 Fig — These graphs show the ordered −log10 of p-values distributions. The first row corresponds to differentiation between controls and two tumour subtypes combined together based on all 48,803 probes from the METABRIC data set. The second row represents the separation between the two actual subtypes. The first column refers to the comparison between luminal A and B subtypes; the number of probes significantly differentiating between luminal tumours and controls was found to be equal to approximately 10,000 (red mark in the top left graph), and 1,000 probes out of the previously defined 10,000 were found to distinguish between the luminal A and B subtypes the most (red mark in the bottom left graph). The results of the comparison between luminal A and basal-like subtypes are shown in the second column. The upper graph corresponds to the separation between luminal A and basal-like tumours put together against the controls. The plot at the bottom shows the Wilcoxon test p-values distributions of the differentiation between luminal A and basal-like tumours. The number of probes in each graph was kept equal to those defined in luminal A and B separation: 10,000 and 1,000 respectively. The third column corresponds to the comparison between luminal A and HER2-enriched, using an analogous procedure as described above. (TIFF) [file pone.0158259.s004.tiff]

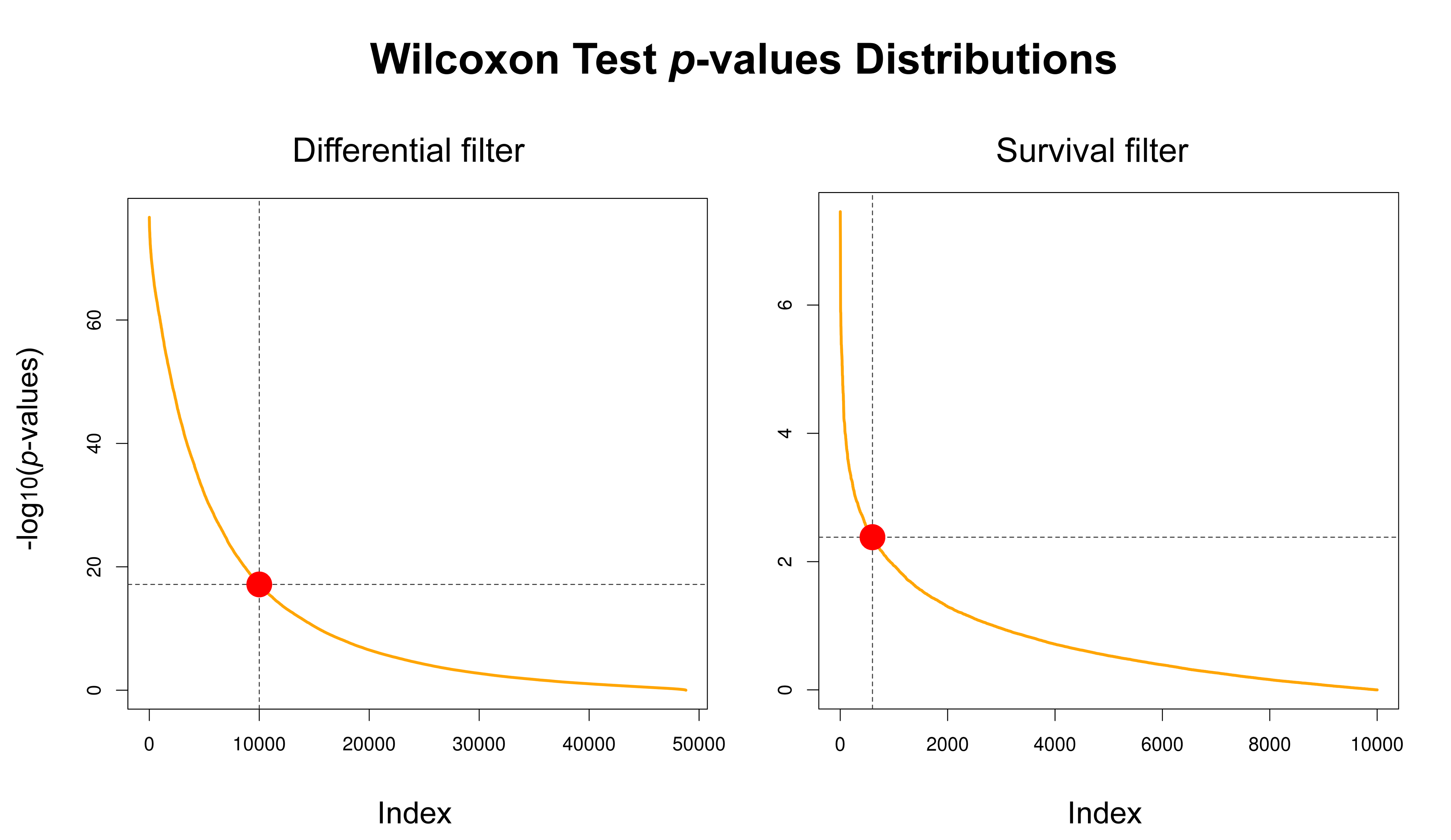

Supplement: S7 Fig — The graph on the left shows the distribution of −log10-normalised p-values, calculated using the Wilcoxon test comparing the expression of ordinary-luminal tumours to control samples. The distribution of p-values calculated using the Log-rank test applied to Kaplan-Meier survival curves, as explained in section Survival Filter, is plotted on the right. (TIFF) [file pone.0158259.s007.tiff]

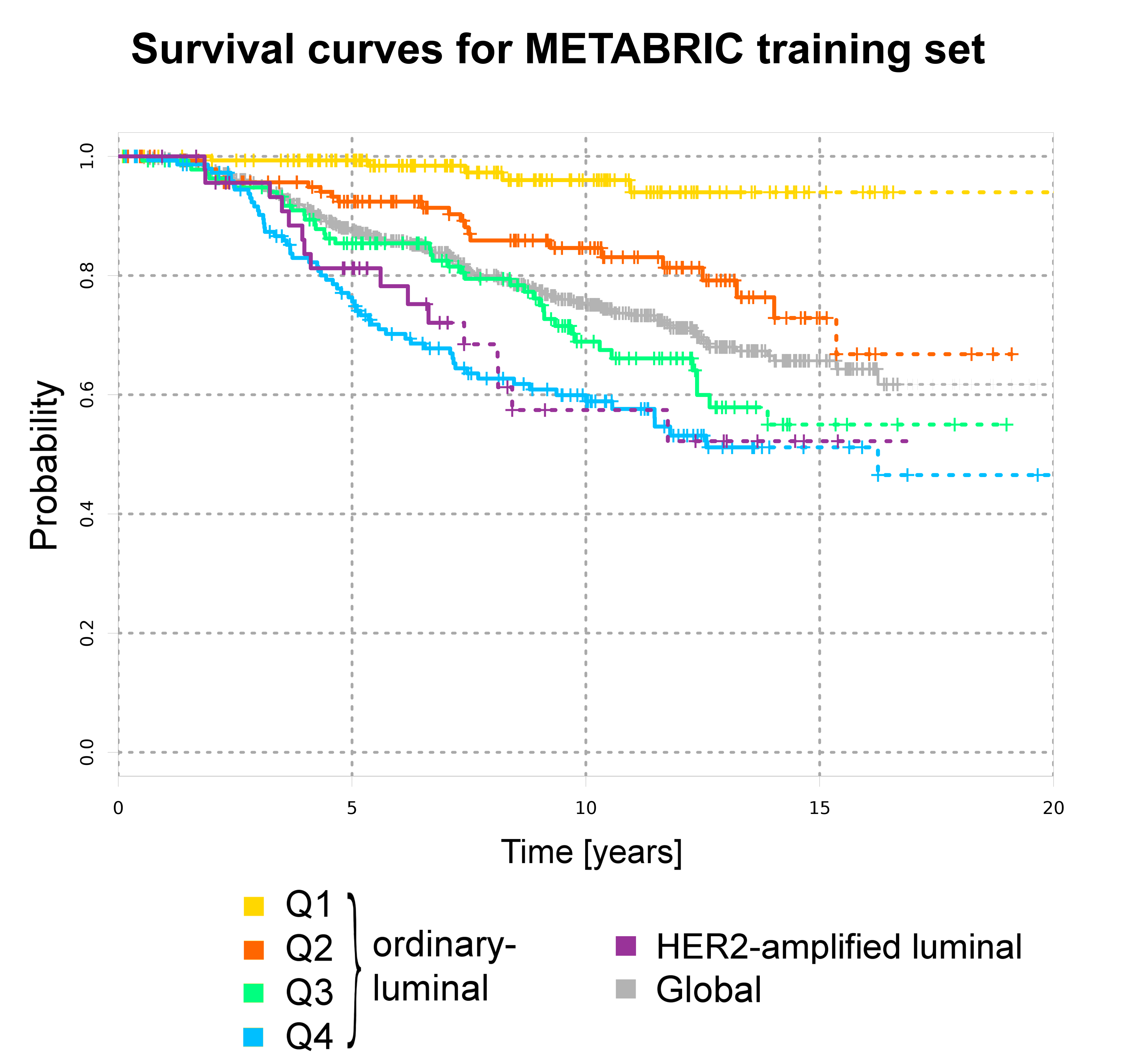

Supplement: S8 Fig — The disease-specific survival probabilities of ordinary-luminal Q1 (yellow), Q2 (orange), Q3 (green) and Q4 (blue), and HER2-amplified luminal (purple) subgroups, stratified based on 600 Survival filter passing probes, in the METABRIC training set (n = 635) are plotted using the Kaplan-Meier estimator. The overall survival rates are shown in grey. Ticks represent sensors, corresponding to patients alive at a given point of time, and the drops represent deaths. The last 20 observations are denoted with a dash line. (TIFF) [file pone.0158259.s008.tiff]
